# Prussian blue-based theranostics for ameliorating acute kidney injury

**DOI:** 10.1186/s12951-021-01006-z

**Published:** 2021-09-06

**Authors:** Dong-Yang Zhang, Hengke Liu, Kathy S. Zhu, Ting He, Muhammad Rizwan Younis, Chen Yang, Shan Lei, Jiayingzi Wu, Jing Lin, Junle Qu, Peng Huang

**Affiliations:** 1 Marshall Laboratory of Biomedical Engineering, International Cancer Center, Laboratory of Evolutionary Theranostics (LET), School of Biomedical Engineering, Shenzhen University Health Science Center, Shenzhen, 518060 China; 2 Key Laboratory of Optoelectronic Devices and Systems of Ministry of Education and Guangdong Province, College of Optoelectronic Engineering, Shenzhen University, Shenzhen, 518060 China; 3 National Clinical Research Center for Oral Diseases, National Engineering Laboratory for Digital and Material Technology of Stomatology, Beijing Key Laboratory of Oral Digital Medicine, Peking University School and Hospital of Stomatology, Beijing, 100081 China

**Keywords:** Prussian blue nanoparticles, Nanozyme, Reactive oxygen/nitrogen species scavenging, Acute kidney injury, Theranostics

## Abstract

**Background:**

Acute kidney injury (AKI) with high mortality rates is associated with an excess of reactive oxygen/nitrogen species (RONS) within kidney tissues. Recently, nanomedicine antioxidant therapy has been used to alleviate AKI. Herein, we synthesized ultrasmall Prussian blue nanozymes (PB NZs, 4.5 nm) as theranostic agents for magnetic resonance (MR)/photoacoustic (PA) dual-modal imaging guided AKI treatment.

**Results:**

PB NZs exhibited multi-enzyme mimetic abilities, promoting the effective elimination of RONS both in vitro and in vivo. Moreover, benefiting from their imaging contrast properties, the rapid renal accumulation of PB NZs was verified by in vivo PA/MR dual-modal imaging. Due to their excellent enrichment in the kidney and unique multi-enzyme mimetic abilities, ultrasmall PB NZs displayed superior AKI treatment efficacy compared with that of amifostine in two clinically relevant types of AKI induced murine models (either by rhabdomyolysis or cisplatin).

**Conclusion:**

Our findings suggested ultrasmall PB NZs, as nanozyme theranostics, have great potential for AKI management.

**Graphic abstract:**

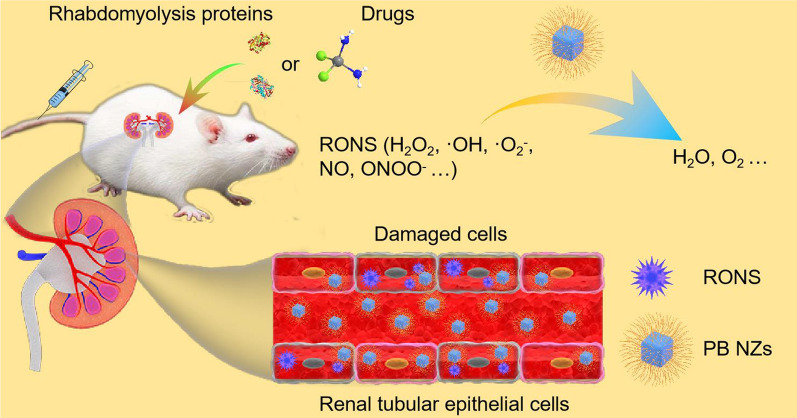

**Supplementary Information:**

The online version contains supplementary material available at 10.1186/s12951-021-01006-z.

## Introduction

Acute kidney injury (AKI), which is characterized by a rapid decline in kidney function, is an important health concern owing to its high morbidity and mortality, with an estimated 1.7 million deaths per year worldwide [1, 2]. At present, adjuvant therapy and kidney transplantation are the most common methods used to treat AKI [3–5]. Recent studies have shown that AKI pathogenesis is related to an excess of intracellular reactive oxygen/nitrogen species (RONS) [6–10]. Previously, some small molecules, e.g., amifostine (AMF) and *N*-acetyl cysteine, have been used as antioxidants that remove reactive oxygen species (ROS) for alleviating AKI symptoms [11, 12]. However, the side effects and limited efficacy of these drugs hinder their clinical applications [13, 14].

Besides, some nanomedicines, such as DNA origami, polyoxometalates, melanin self-assembled nanoparticles (NPs), black phosphorus nanosheets, and selenium-doped carbon quantum dots, have also been developed to alleviate AKI by consuming overabundant ROS [15–23]. Recently, nanozymes with multienzyme-like activities have attracted widespread attention because of prominent advantages and widely applied in the treatment of tumor and ROS-related diseases, which can continuously catalyze the removal of RONS [24–33]. For example, Liu et al. described ultrasmall Cu-based nanozymes (NZs) with multiple enzyme-mimicking and broad-spectrum ROS scavenging abilities for the treatment of inflammation related diseases [33]. As a US Food and Drug Administration (FDA)-approved drug for clinical applications, Prussian blue (PB) NPs as NZs have also been widely explored in biomedical applications [34–39]. For example, PB nanozymes (PB NZs) have exhibited catalase (CAT)-like activity and therefore catalyzed the breakdown of hydrogen peroxide (H_2_O_2_), producing oxygen in tumor microenvironments for photodynamic therapy [35]. In addition, PB NZs can be detected via photoacoustic (PA) and magnetic resonance (MR) imaging, offering the potential for multi-modal imaging guided therapy [36–39]. However, PB NZs have not been explored for AKI management. Furthermore, targeted renal aggregation or excretion could be achieved with ultrasmall size of NPs [40–48]. Therefore, ultrasmall PB NZs with multiple enzyme-like activities could potentially facilitate the removal of RONS in kidney under imaging guidance/monitoring, achieving efficient AKI theranostics [34, 49, 50].

**Scheme 1:**
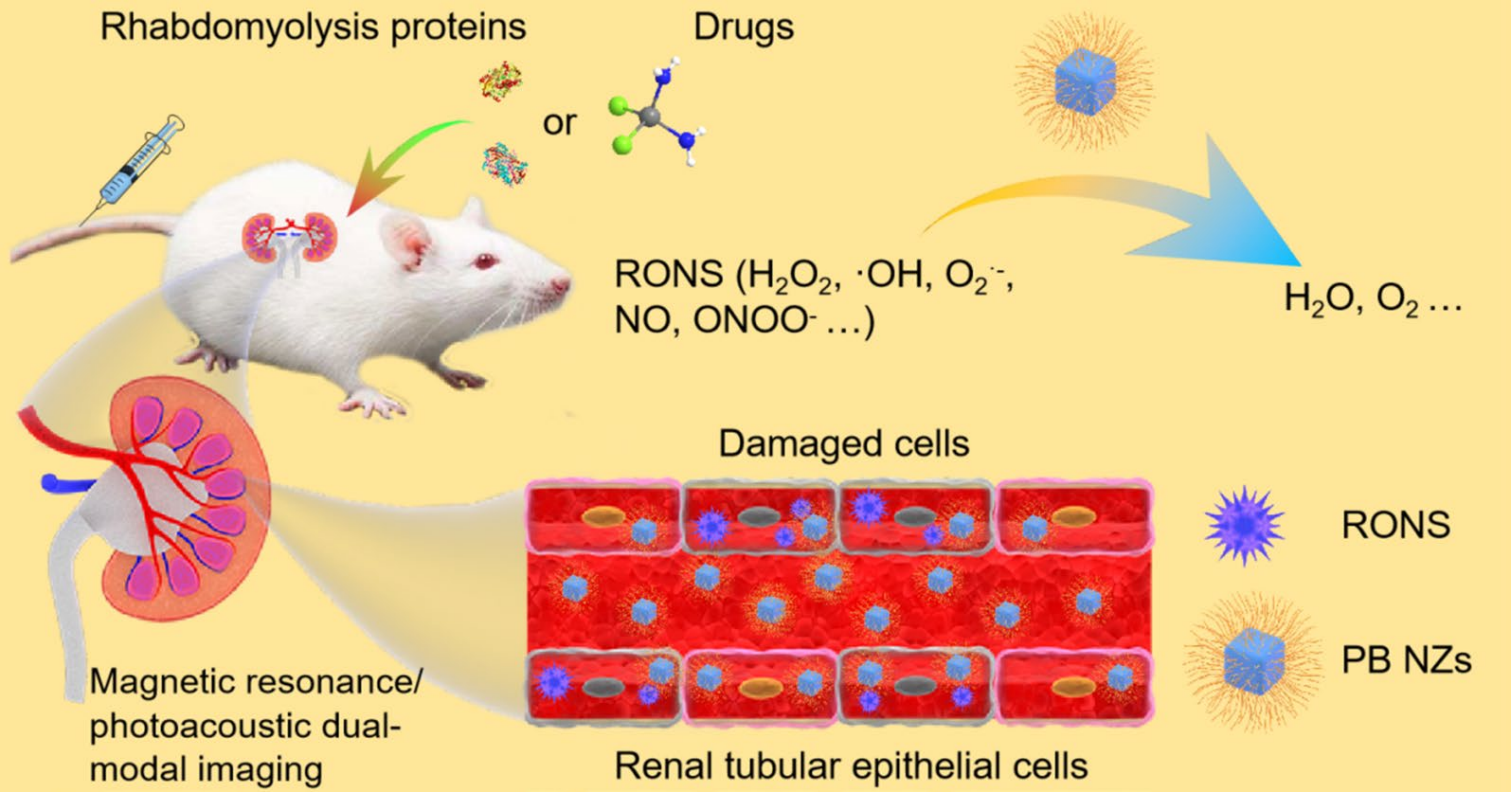
Schematic illustration of Prussian blue nanozymes (PB NZs) as theranostic agent with preferential kidneys enrichment and rapid clearance for magnetic resonance/photoacoustic dual-modal imaging guided acute kidney injury (AKI) treatment through the effective removal of various reactive oxygen/nitrogen species (RONS)

Herein, we used biopolymer chitosan (CS) as a template to synthesize ultrasmall PB NZs for AKI theranostics. CS, a naturally occurring linear cationic polysaccharide with good biocompatibility and biodegradability, and low immunogenicity, has been used as a building block to develop nanomedicine for AKI treatment [51, 52]. As illustrated in Scheme 1, ultrasmall PB NZs were synthesized for MR/PA dual-modal imaging-guided treatment of AKI. The as-prepared PB NZs possess a variety of enzyme-like activities, including CAT, peroxidase (POD), and superoxide dismutase (SOD), which can effectively eliminate various RONS [e.g., H_2_O_2_, hydroxyl radical (·OH), superoxide anion (O_2_^**.**−^), nitric oxide (NO), and peroxynitrite (ONOO^−^)] both in vitro and in vivo. The MR/PA dual-modal imaging capabilities of PB NZs enable visual monitoring of their absorption, distribution, metabolism, and excretion in kidneys. Impressively, the therapeutic effects of PB NZs are far superior to those elicited by the same dose of a commercially available small-molecule drug, AMF. Owing to their ultrasmall size, PB NZs exhibit excellent enrichment in the kidneys and low systemic toxicity, promising for AKI management.

## Material and methods

### Synthesis of PB NZs

First, 300 mg CS (100–300 k) powder was dissolved in 100 mL hydrochloric acid (0.5 mol/L) aqueous solution, containing a small amount of acetic acid. The solution was stirred overnight at room temperature and then filtered through a 0.45 μm filter. K_3_Fe(CN)_6_ aqueous solution (20 mL, 1 mM) was added to 80 mL of the CS solution and placed on a magnetic stirrer. After 0.5 h, FeCl_2_ solution (20 mL, 1 mM) was slowly dropped into the mixture over 10 min. After 1 h, acetone (200 mL) was added, and the mixture was centrifuged at 10,000 rpm for 15 min. The precipitates were washed three times with acetone. Finally, the PB NZs powder sample was obtained by vacuum drying at 50 °C. The obtained solid sample was dissolved in phosphate buffered saline (PBS) and the iron content was quantified by inductively coupled plasma mass spectrometry (ICP-MS).

### Animals and the rhabdomyolysis- or cisplatin-induced AKI models

All animal studies were approved by the Animal Ethics and Welfare Committee of Shenzhen University. Female BALB/c mice were purchased from Guangdong Medicinal Laboratory Animal Center (Guangzhou, China) Mice aged 4–6 weeks with body weights of 18–21 g were used in all animal studies. Distilled water and sterile food were provided to all mice. Animals were acclimatized to their new environment for 5 days prior to treatment.

The mice were used to establish either rhabdomyolysis-induced (RM-)/cisplatin-induced (CP-) AKI models, as follows. (i) RM-AKI model. Female BALB/c mice were deprived of water for 15 h but had access to food. At the end of the water restriction, 8 mL/kg of 50% glycerol was *i.m.* injected to each hind limb of the mice. Then, all mice were given free access to water and food. After 24 h, the mice were euthanized, and the blood and renal tissues were collected and analyzed. (ii) CP-AKI model. Female BALB/c mice were *i.p.* injected with 20 mg/kg cisplatin. After 3 days, the mice were euthanized, and the blood and renal tissues were collected and analyzed.

### In vitro and in vivo measurements of MR/PA imaging

For in vitro* T*_2_-weighted MR imaging, a series of PB NZs aqueous solutions of different concentrations (0.56, 0.112, 0.225, 0.45, 0.9 and 1.8 mg/mL; 0.056, 0.112, 0.225, 0.45, 0.9, and 1.8 mM Fe) were evaluated and the *r*_2_ co-efficient was calculated.

For in vivo* T*_2_-weighted MR imaging, healthy or RM-AKI mice (*n* = 3) were *i.v.* injected with 100 µL of PB NZs solution in PBS (2 mg/mL). Subsequently, the mice were imaged at different time points (0, 0.5, 1, 2, 4, and 8 h) *p.i.*, using a 4.7 T small animal scanner (United Imaging, China).

PA imaging of the kidneys of RM-AKI mice prior to PB NZs injection was performed as a control, using a PA imaging instrument (Vevo LAZR2100, VisualSonics, Canada). The RM-AKI mice were then anesthetized and given an *i.v.* injection of PB NZs (200 μL, 2 mg/mL) in PBS (*n* = 3). The PA imaging instrument was then used to obtain PA images of the kidneys from RM-AKI mice at various time points (0, 2, 4, and 8 h) *p.i*.

### Treatment of AKI mice

Different treatments were administered to the AKI model mice, as follows. (i) Healthy mice were treated with PBS (*n* = 5) or PB NZs (100 μg in 100 μL PBS, *n* = 5); (ii) RM-AKI mice were treated with PBS (*n* = 5), AMF (100 μg in 100 μL PBS, *n* = 5), or PB NZs (100 μg in 100 μL PBS, *n* = 5); (iii) CP-AKI mice were treated with PBS (*n* = 5), AMF (100 μg in 100 μL PBS, *n* = 5), or PB NZs (100 μg in 100 μL PBS, *n* = 5). In the RM-AKI mouse model, treatment groups were *i.v.* injected following *i.m*. injection of 50% glycerol. Treatment groups were *i.v.* injected 30 min (AMF) or 4 h (all others) prior to intraparietal injection of cisplatin into CP-AKI mice. Their blood and kidney function indicators were compared with healthy control mice after 24 h *p.i.* (RM-AKI) or 72 h *p.i.* (CP-AKI). The survival curves and body weights of AKI mice were monitored for 14 days after treatment. According to animal welfare guidelines, mice with a weight loss of more than 15% were considered dead in survival curves.

The mice were euthanized, then blood samples were collected and centrifuged at 5000 rpm for 10 min at 4 °C. The concentration of blood urea nitrogen (BUN) and creatinine (CRE) in the serum samples was determined by Wuhan Servicebio Technology Co., Ltd. Organs collected from the mice were fixed with 4% paraformaldehyde, and the samples were stained with Hematoxylin and eosin (H&E) for histological analysis.

### Analysis of renal tissues after treatment

Kidneys collected from each group were stored in a refrigerator at − 80 °C. Kidney homogenate supernatants were prepared according to different assay protocols. SOD levels were measured using a SOD assay kit (Sigma-Aldrich, USA). Heme oxygenase-1 (HO-1) and Kidney injury molecule-1 (KIM-1) expression levels were determined using HO-1 and KIM-1 ELISA kits (Abcam, USA), respectively. The degree of DNA damage was assessed using a DNA damage competitive enzyme linked immunosorbent assay (ELISA) kit (Invitrogen, USA). The level of lipid peroxidation was assessed using a thiobarbituric acid-reactive substances (TBARS) assay kit (Cayman Chemical, USA). The level of RNS was measured by a tissue RNS detection kit (Rhein, China).

### In vivo toxicity evaluation and metabolism

Healthy mice were i.v. injected with either PB NZs (50 mg/kg in 150 µL) or PBS (150 µL) as controls. The hematological parameters aspartate aminotransferase (AST), alanine aminotransferase (ALT), BUN, and CRE were determined 14 days *p.i.* of PB NZs into mice. The major organs (heart, liver, spleen, lungs, and kidneys) collected from each group were stained with H&E. Mice body weights were recorded every 2 days.

The mice were intravenously administered with PB NZs (100 μg in 100 μL PBS, *n* = 3) and the urea was collected at different time point (12 and 24 h). The urine was digested by aqua regia and diluted to detect iron content by ICP-MS.

### Statistical analysis

Representative results are presented in this report. Quantitative data are presented as mean ± standard deviation (SD). *P*-values less than 0.05 were considered as represent a statistically significant difference between groups.

## Results and discussion

### Synthesis and characterization of PB NZs

**Fig. 1:**
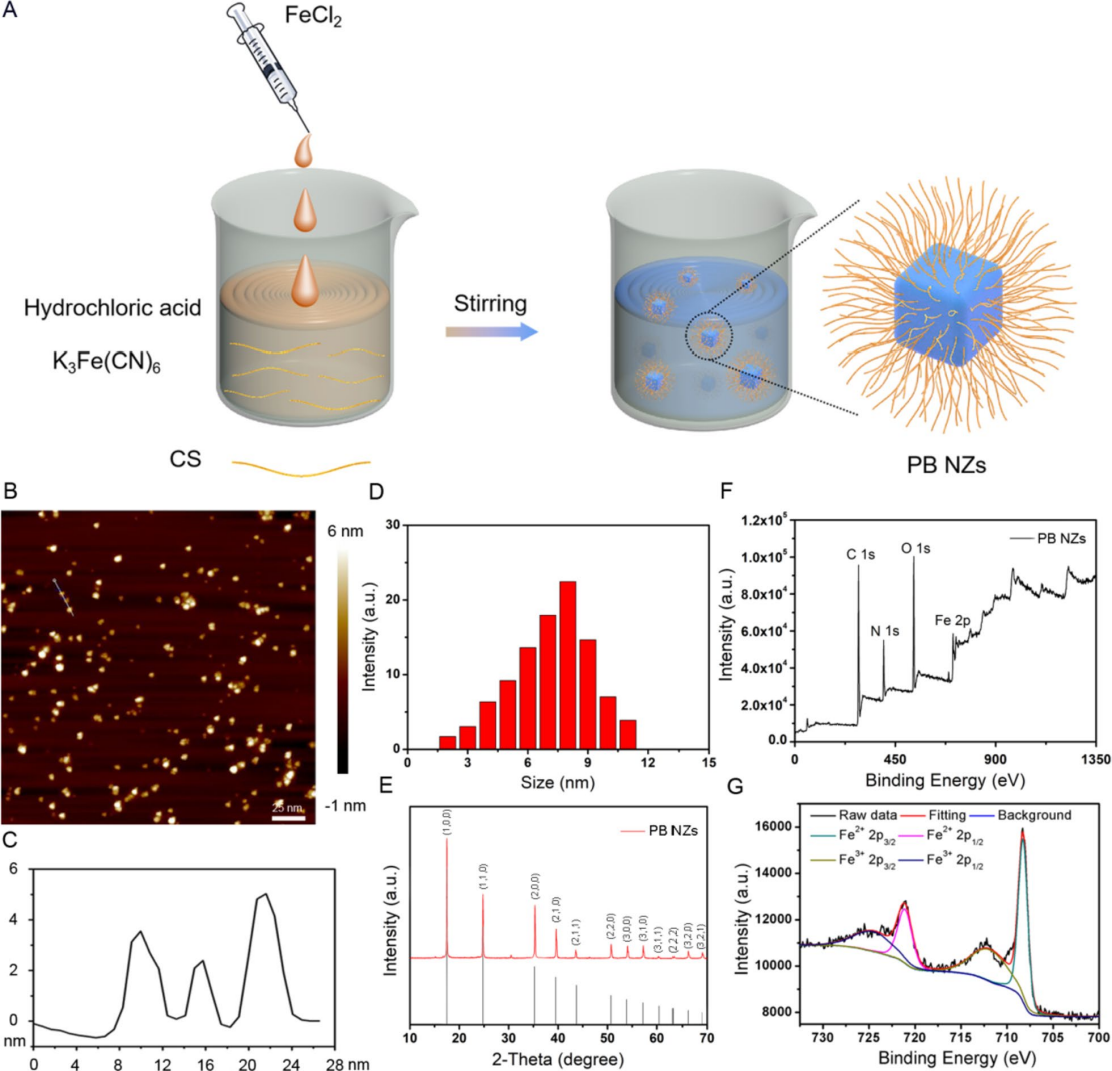
**A** Schematic illustration of the preparation of PB NZs. **B** AFM image of PB NZs. The scale bar is 25 nm. **C** The corresponding height image of selected nanoparticles. **D** Size distribution of PB NZs in PBS. **E** XRD pattern of PB NZs. **F** XPS and **G** Fe 2p XPS spectra of PB NZs

The PB NZs were synthesized by a simple solution reaction using CS as a template (Fig. 1A). The atomic force microscopy (AFM) image revealed an average size of PB NZs is about 4 nm and a thickness of approximately 3.5 nm (Fig. 1B, C), which is in accordance with the dynamic light scattering (DLS) measurement (Fig. 1D). The morphology and size of PB NZs were also characterized by transmission electron microscopy (TEM), indicating spherical shape PB NZs with about 3–4 nm diameter (Additional file 1: Fig. S1). As shown in Fig. 1E, X-ray powder diffraction (XRD) pattern confirmed that the structure of PB NZs can be indexed to the face-centered cubic lattice of Fe_4_[Fe(CN)_6_]_3_ nanocube according to JCPDS No. 0-0239. The results of X-ray photoelectron spectroscopy (XPS) demonstrated the presence of Fe elements and two ionic forms, Fe^3+^ and Fe^2+^ (Fig. 1F, G), with a molar ratio of approximately 1.3:1 between Fe^2+^ and Fe^3+^. Furthermore, Fourier-transform infrared (FTIR) spectra indicated the presence of a C=O bond peak at 1628 cm^−1^, confirming the successful coating of CS (Additional file 1: Fig. S2). As shown in Additional file 1: Fig. S3A, B, the PB NZs exhibited good solubility and physiological stability in different biological media, e.g., PBS, DMEM, and FBS for several days. The absorbance spectra of aqueous suspensions of PB NZs demonstrated strong NIR absorption at 700 nm, while the molar extinction co-efficient of the corresponding metal atom (Fe) was 6080 cm^−1^ M^−1^ at 700 nm (Additional file 1: Fig. S3C), indicating the potential of PB NZs as a PA contrast agent. Finally, the proportion of PB (70%) and CS to PB NZs (12%) was detected by thermogravimetric analysis (TGA, Additional file 1: Fig. S4) and ICP-MS.

### Scavenging of RONS by PB NZs

**Fig. 2:**
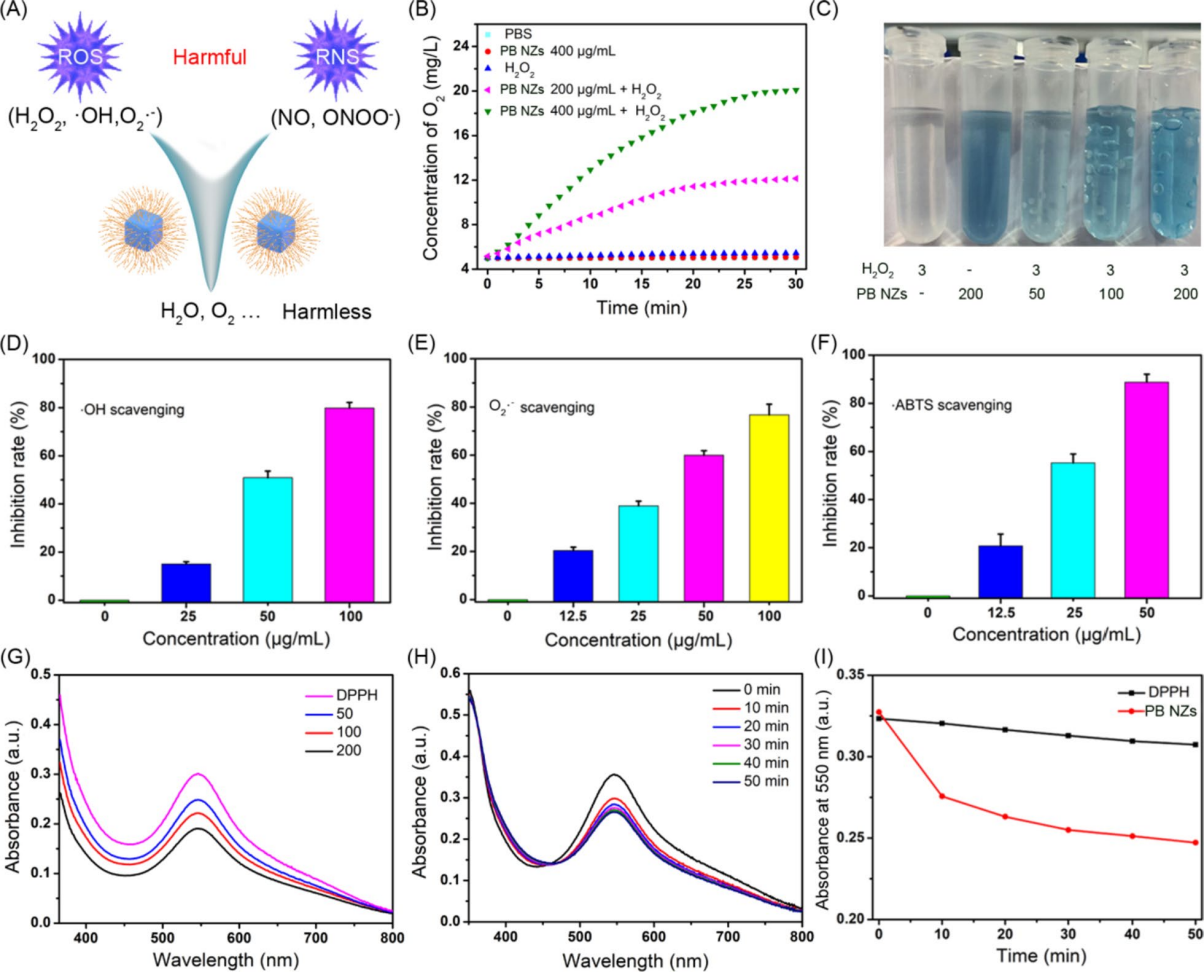
**A** Illustration of RONS scavenging by PB NZs. **B** O_2_ generation by different indicated sample solutions at 37 °C. **C** The production of O_2_ bubbles in different solutions after reaction for 10 min in PBS (pH 7.4). The units of H_2_O_2_ and PB NZs are wt% and µg/mL, respectively. **D** ·OH scavenging ability of PB NZs at different concentrations. **E** O_2_^**.**−^ free radical scavenging ability of PB NZs at different concentrations. **F** Free radical-scavenging ability of PB NZs at different concentrations evaluated by an ABTS assay. **G** Antioxidant ability of PB NZs against different concentrations of DPPH. **H** Absorption spectra of DPPH solution with 200 µg/mL PB NZs added, from 0 to 50 min. **I** Time-dependent absorbance at 550 nm of DPPH alone or treated with 200 µg/mL PB NZs

Based on the previous literatures [34, 35], we systematically investigated the potential multienzyme mimetic abilities of PB NZs for removing a variety of RONS (Fig. 2A). First, O_2_ generation was verified using a dissolved O_2_ electrode, which showed O_2_ gradually increased with time and the concentration of PB NZs (Fig. 2B). Simultaneously, O_2_ bubbles were only produced in solution in the presence of both PB NZs and H_2_O_2_, while at higher concentration of PB NZs, more O_2_ generation was noticed, as shown in Fig. 2C. The O_2_ content in the solution was further detected by using the Tris (4,7-diphenyl-1,10-phenanthroline) ruthenium (II) dichloride (Ru(dpp)_3_Cl_2_) complex as an oxygen probe, because O_2_ quenches the phosphorescence of the Ru-complex. Additional file 1: Fig. S5 showed that the phosphorescence of Ru-complex was greatly reduced in response to increasing concentrations of H_2_O_2_, confirming the CAT-like activity of PB NZs. Next, we investigated the POD-like activity of PB NZs in PBS (pH 7.4) at room temperature using steady-state kinetic analysis. The enzymatic parameters were calculated using the Michaelis–Menten equation and a Lineweaver–Burk plot (Additional file 1: Fig. S6), which showed *K*_m_ values of 1.4 mM and 20.7 mM for tetramethyl benzidine (TMB) and H_2_O_2_, respectively. Subsequently, ESR assay and a ·OH assay kit were used to evaluate the POD-like activity of PB NZs. The characteristic peaks of ·OH and 5,5-dimethyl-1-pyrroline *N*-oxide (DMPO) adducts were weaken in ESR assay, which indicated that PB NZs could effectively scavenge ·OH (Additional file 1: Fig. S7A). Notably, a concentration-dependent scavenging property of PB NZs toward ·OH was noticed as approximately 79.8 ± 2.4% of ·OH were scavenged at a concentration of 100 µg/mL (Fig. 2D). Similarly, the characteristic peaks of O_2_^**.**−^ and DMPO adducts were also weaken in ESR assay, which further confirmed that PB NZs could also effectively scavenge O_2_^**.**−^ (Additional file 1: Fig. S7B). More than 75% of O_2_^**.**−^ were scavenged by 100 µg/mL of PB NZs, as estimated using a commercial SOD assay kit (Fig. 2E). The widely antioxidative properties of PB NZs were further confirmed using a 2,2′-azino-bis(3-ethylbenzothiazoline 6-sulfonate radical (·ABTS) assay. Figure 2F showed an elimination of 88.8 ± 3.3% of free radicals at 50 μg/mL of PB NZs. As with ROS, excessive reactive nitrogen species (RNS) can also induce renal cell damage, resulting in AKI [53]. Therefore, we also investigated the RNS scavenging ability of PB NZs using the 2,2-di-(4-*tert*-octylphenyl)-1-picrylhydrazyl (DPPH) free radical [54]. The results showed an effective elimination of DPPH with time- and concentration-dependent properties (Fig. 2G–I), indicating outstanding RNS scavenging efficiency. Taken together, these results demonstrated that PB NZs possess impressive antioxidant activity against various RONS in vitro.

### ***PB NZs mediate protection of H***_***2***_***O***_***2***_***-stimulated cells***

**Fig. 3:**
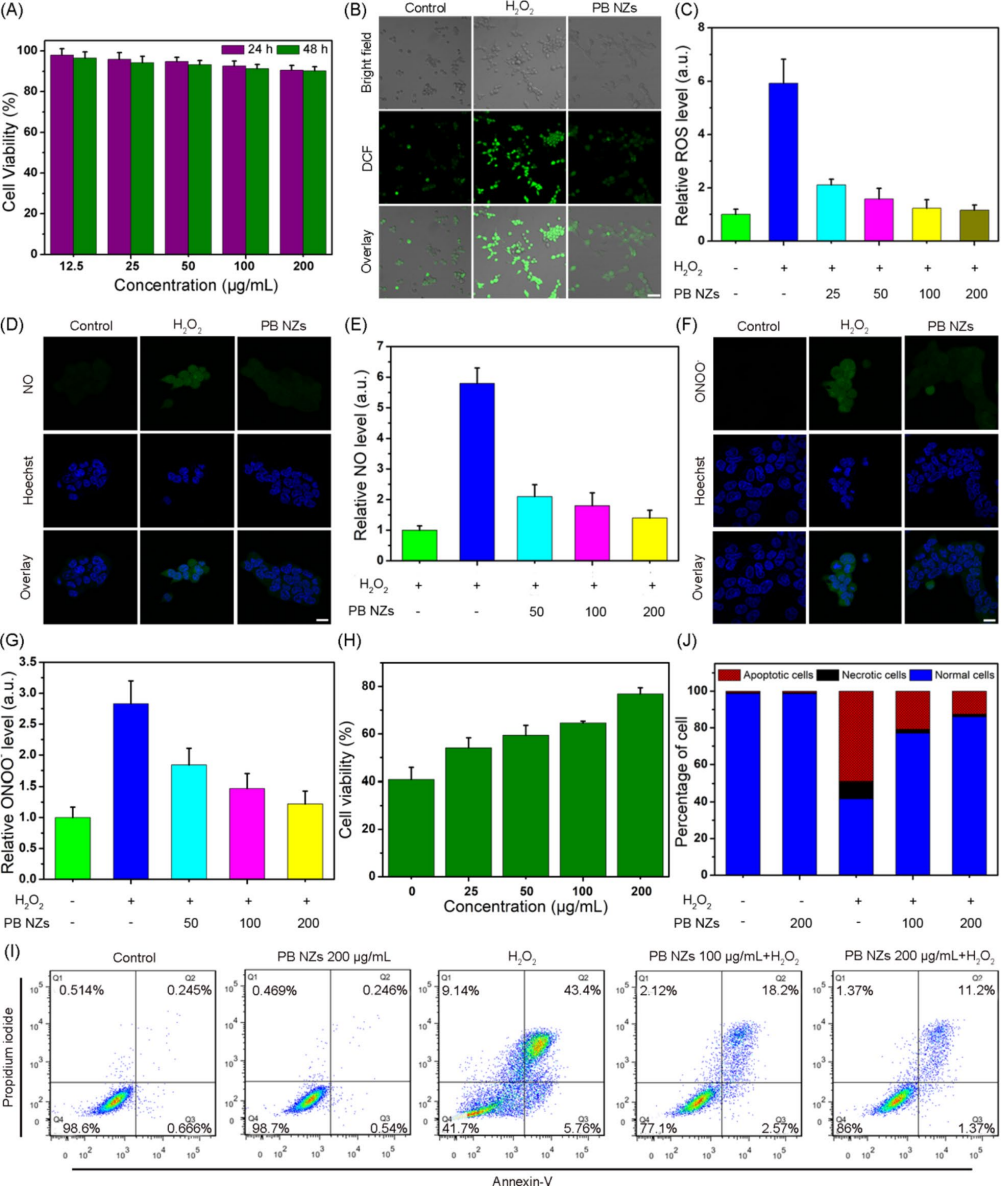
**A** The viability of HEK239T cells treated with PB NZs for 24 and 48 h, respectively. **B** Representative confocal fluorescence microscopy images of cellular ROS levels detected by DCFH-DA staining under different indicated treatment conditions. The scale bar is 20 μm. **C** Relative ROS levels in HEK293T cells incubated with 0.5 mM H_2_O_2_ under different indicated treatment conditions. Fluorescence images (**D**) and corresponding statistical histograms (**E**) of intracellular NO levels under different conditions. The scale bar is 5 μm. Fluorescence images (**F**) and corresponding statistical histograms (**G**) of intracellular ONOO^−^ levels under different conditions. The scale bar is 5 μm. **H** Viability of HEK293T cells after treatment with 0.5 mM H_2_O_2_ or different concentrations of PB NZs. A scatter diagram (**I**) and a histogram (**J**) of cell apoptosis and necrosis distribution in untreated and PB NZs-treated HEK293T cells

To consider the biological applications of PB NZs, their biocompatibility was evaluated using MTT and hemolysis assay. No apparent cytotoxicity was observed at increasing concentrations of PB NZs up to 200 μg/mL, as the survival rate of HEK293T cells was > 85% (Fig. 3A). Additionally, a hemolysis rate of less than 5% was seen at a higher concentration of PB NZs (5 mg/mL), indicating their excellent biological safety (Additional file 1: Fig. S8). Excessive quantities of RONS cause tubular damage that can lead to AKI; therefore, HEK293T cells were used to determine the protective effect of PB NZs against RONS-induced damage. Compared with cells stimulated by H_2_O_2_, representative confocal fluorescence microscopy images of DCF staining showed significantly lower ROS levels in HEK293T cells pre-treated with PB NZs, which is comparable with untreated cells (Fig. 3B). Moreover, when the cells were pre-treated with different concentrations of PB NZs, the levels of intracellular ROS were significantly reduced, in a concentration-dependent manner, compared with ROS levels in the untreated group (Fig. 3C), suggesting PB NZs have excellent ROS scavenging ability. Similarly, H_2_O_2_ stimulation led to strong fluorescence and produced large quantities of RNS, while in comparison with the control group, the cells pre-treated with PB NZs significantly reduced intracellular NO and ONOO^−^ (Fig. 3D–G), as monitored by DAF-FM DA and DAX-J2™ PON Green staining, respectively. Given that RONS can interfere with mitochondrial function and cause cell death due to oxidative stress [55, 56], PB NZs clearly reduced such responses and offered appreciable cellular protection from RONS (Additional file 1: Fig. S9). Finally, the results of MTT (Fig. 3H) and apoptosis detection (Fig. 3I, J) assays revealed that H_2_O_2_ stimulation could induce apoptotic and necrotic cells death via the production of RONS, while the cell survival rate improved remarkably following pre-treatment with PB NZs, in a concentration-dependent manner.

### Dual-modal MR/PA imaging of PB NZs in mice with AKI

**Fig. 4:**
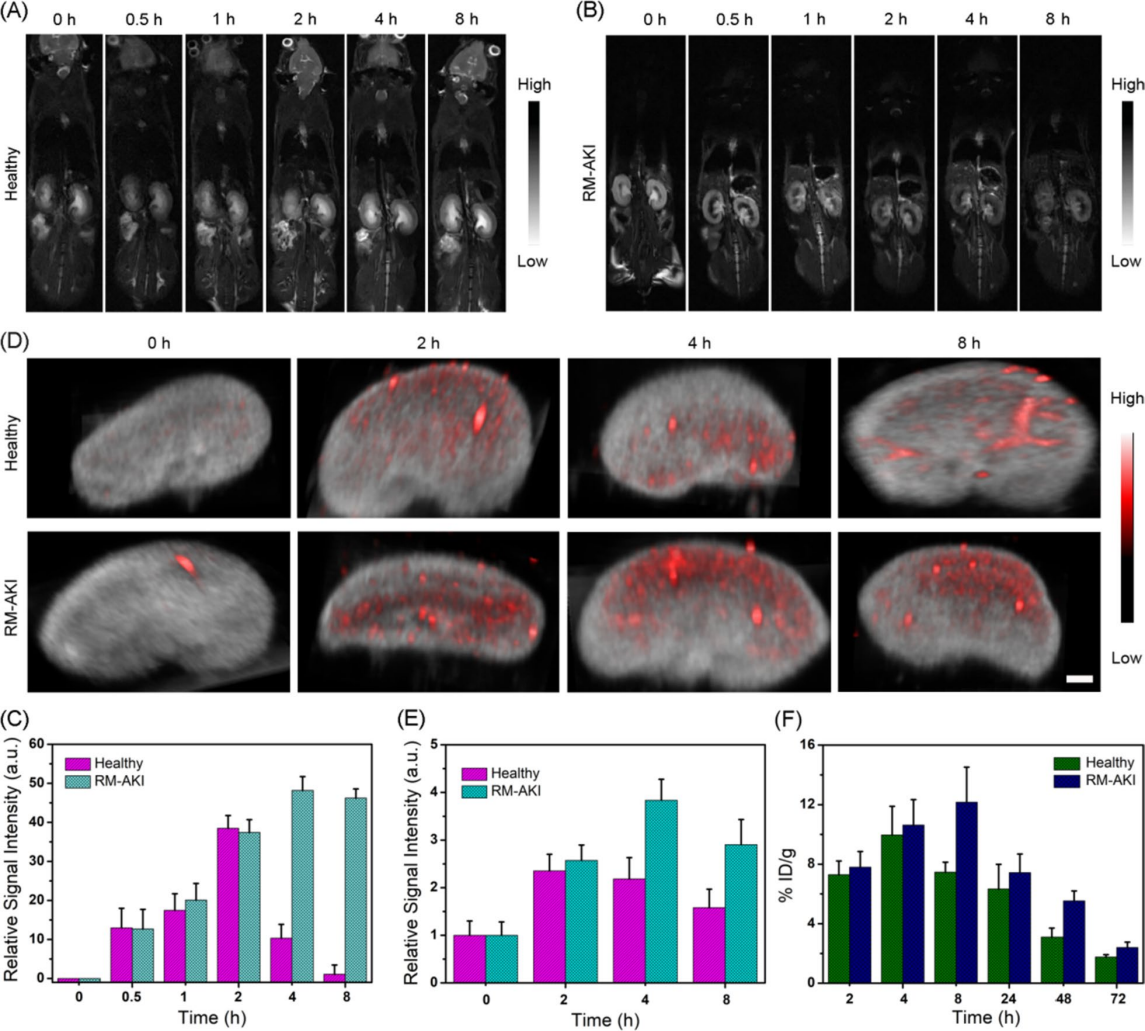
In vivo imaging and distribution of PB NZs in healthy and AKI model mice. *T*_2_-weighted MR images of PB NZs in **A** healthy and **B** RM-AKI mice at pre-injection (0 h) and different post-injection time points. The injection dose of PB NZs (2 mg/mL) is 200 μL. **C** Quantitative analysis of the *T*_2_-weighted MR signal-intensity rates in the kidneys of healthy and RM-AKI mice. (*n* = 3; mean ± SD). **D** Three-dimensional (3D) photoacoustic (PA) images and **E** relative PA signal intensities in the kidneys of healthy and RM-AKI mice at different time points following *i.v.* injection of PB NZs. The injection dose of PB NZs (2 mg/mL) is 200 μL. The scale bar is 1 mm. **F** Time-dependent accumulation of PB NZs in the kidneys of RM-AKI and healthy mice as measured by ICP-MS. The injection dose of PB NZs (2 mg/mL) is 200 μL

Owing to their potential capability of the simultaneous diagnosis and treatment of diseases, theranostic agents have become the subject of emerging research in recent years [57–61]. However, multimodal imaging is a much better and more suitable option for precise diagnosis than a single imaging modality, as the former can overcome the limitations of the latter by integrating two or more imaging patterns [62, 63]. As shown in Additional file 1: Fig. S10, the *T*_2_-weighted MR images of PB NZs became darker with an increase in the concentration of PB NZs. The transverse relaxivity rate (*r*_2_) of PB NZs was calculated to be 54.1 mM^−1^ S^−1^. First, an intramuscular (*i.m.*) injection of 50% glycerol was administered to dehydrated healthy mice and establish a murine AKI model (Additional file 1: Fig. S11). Next, in vivo MR imaging was performed, due to its advantages of high resolution and no depth limitation [64, 65]. Figure 4A, B show significantly enhanced *T*_*2*_-weighted MR signals in the kidneys of healthy and rhabdomyolysis (RM)-AKI mice following intravenous (*i.v.*) injection of PB NZs. A quantitative analysis of the MR images revealed an approximately 1.38-fold higher MR signal (at 2 h) than prior to the injection of PB NZs (Fig. 4C). Similarly, the *T*_*2*_-weighted MR images showed a profound increase in renal signals, with a maximum enhancement of 48% in the quantitative signal-to-noise ratio (SNR) at 4 h post-injection (*p.i.*), suggesting that PB NZs stayed longer in the kidneys of AKI mice than healthy mice [66].

With their high absorption in the near-infrared (NIR) region, PB NZs hold great potential for PA imaging, which can be further exploited to evaluate their effective accumulation in the kidneys [67, 68]. Additional file 1: Fig. S12A, B showed that a gradual increase in PB NZs concentration led to a more obvious PA signal, indicating a good PA imaging capacity. On the other hand, negligible PA signals were noticed in the kidneys prior to the injection of PB NZs, whereas PA signals were remarkably enhanced after 4 h *p.i*^.^ in the kidneys of RM-AKI mice, with an approximate enhancement of 3.8-fold under 700 nm laser irradiation (Fig. 4D, E). Similarly, PA signals in the kidneys of healthy mice after 2 h *p.i* were 2.3-fold stronger than prior to the injection of PB NZs. The blood circulation curve in Additional file 1: Fig. S13 indicated that the blood pharmacokinetics of PB NZs comprised a typical two-compartment model and the half-life of the blood distribution phase was around 3.63 h, suggesting relatively rapid blood elimination in vivo. The biodistribution of PB NZs following their *i.v.* administration in mice was evaluated by an ICP-MS experiment, which revealed 15.3% and 6.3% ID/g accumulation in the liver and kidneys, respectively (Additional file 1: Fig. S14). It is either superior or similar to other reported nanomaterials (Additional file 1: Table S1). However, rapid excretion from the kidneys was observed at 72 h (Fig. 4F), suggesting good biological safety of PB NZs. Meanwhile, the amount of iron ion in urine was detected, proving that PB NZs can be excreted through urine metabolism (Additional file 1: Fig. S15). These results are in good agreement with the MR imaging, and thus verified the rapid and effective accumulation of PB NZs in the kidneys, offering sufficient guidance for AKI treatment.

### Treatment of murine AKI with PB NZs

**Fig. 5:**
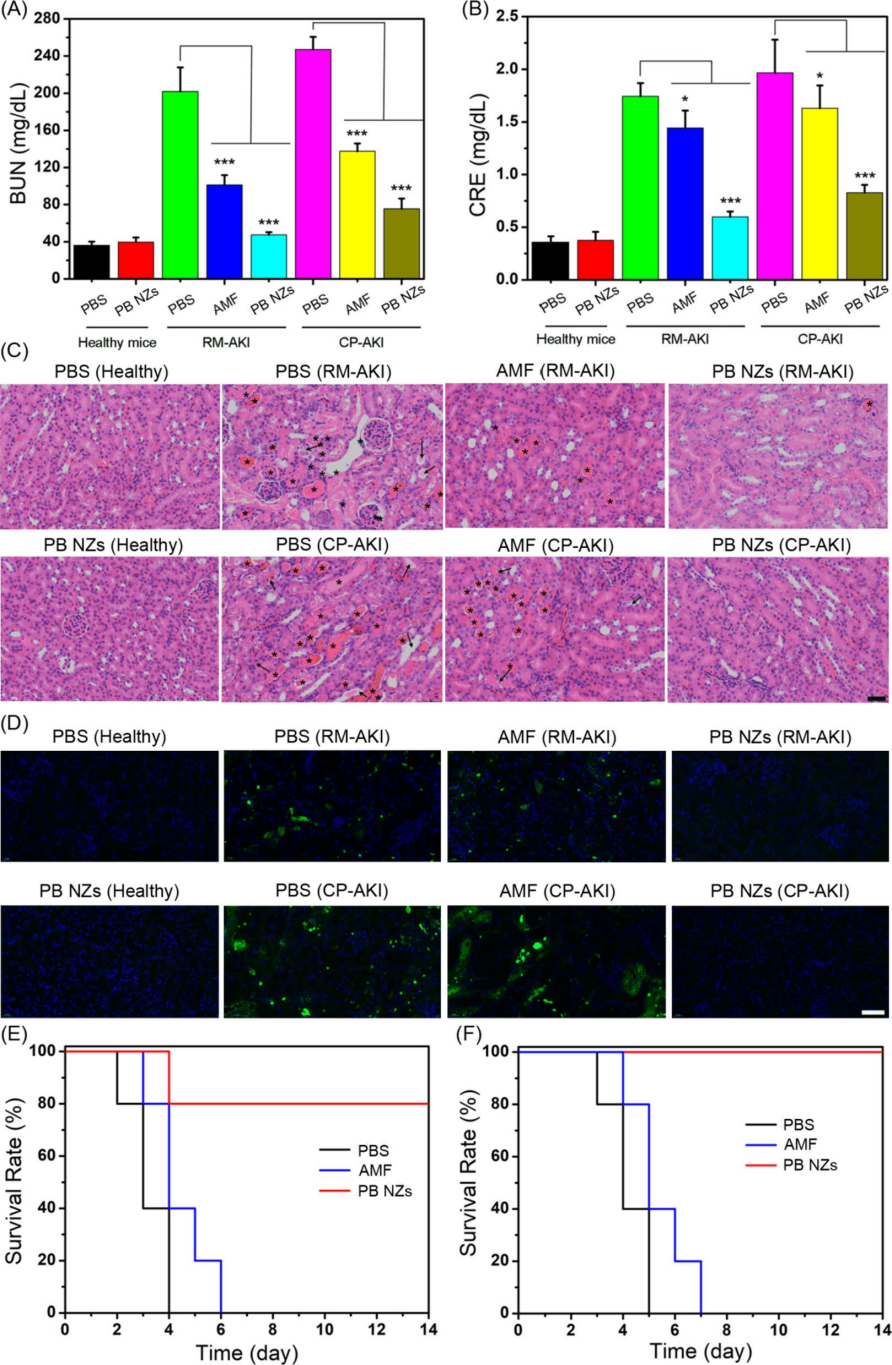
Estimation of kidney function indicators: **A** BUN and **B** CRE in healthy mice and RM-AKI and CP-AKI mice treated with PBS, AMF or PB NZs. Data represent the mean ± SD (*n* = 3). *ns* non-significant, *P < 0.05, **P < 0.01, and ***P < 0.001 versus the RM-AKI or CP-AKI PBS-treated group, respectively. The injection dose of PB NZs (2 mg/mL) is 100 μL. **C** H&E stained images of kidney tissues collected from each group. Arrows indicate damaged tubules, and asterisks indicate the formation of casts. The scale bar is 100 µm. **D** Fluorescence images obtained by the TUNEL assay, with cell nuclei stained with DAPI; green fluorescence indicates cellular apoptosis. The scale bar is 20 µm. The survival curves of **E** RM-AKI and **F** CP-AKI mice in the 14 days following PBS, AMF, or PB NZs treatment. The injection dose of PB NZs (2 mg/mL) is 100 μL

CP-AKI mouse model was established through an intraperitoneal (*i.p.*) injection of cisplatin into healthy mice (Additional file 1: Fig. S16). Based on the in vitro RONS scavenging and in vivo dual-modal MR/PA imaging, the in vivo AKI treatment was investigated on two models of AKI, induced by either rhabdomyolysis or cisplatin. For comparison, AMF, an FDA-approved small-molecule drug for the prevention of CP-AKI, was used as a positive control. First, we measured the levels of clinically important indicators of renal function such as BUN and CRE in serum samples from different groups [69, 70]. Although the PBS groups showed high values for these renal indicators, the PB NZs treated groups exhibited a significant drop in the levels of both BUN and CRE (Fig. 5A, B), suggesting an effective restoration of kidney function. The same dose of NZs was shown to be more effective than AMF. Furthermore, H&E staining of kidney tissues was performed to observe the in vivo AKI therapeutic effect of PB NZs. As shown in Fig. 5C, damaged tubules (marked as arrows) and the formation of casts (marked as asterisks) are present in kidney sections of both PBS-treated RM-AKI and CP-AKI mice, while few damaged structures were found in the PB NZs-treated groups. Cellular apoptosis has been reported to be closely associated with AKI [3]. The fluorescence images from the terminal-deoxynucleotidyl transferase mediated nick end labeling (TUNEL) assay exhibited the highest level of cell apoptosis in kidneys harvested from RM-AKI or CP-AKI mice, while no significant level of apoptosis was found in the PB NZs-treated group (Fig. 5D), suggesting that PB NZs could reduce the level of cell apoptosis in renal tissues. Notably, PB NZs had better treatment efficacy than AMF at the same dose, which is consistent with the results of the blood analysis. The survival curve (Fig. 5E, F), body weight, and H&E staining analysis of kidney tissues (Additional file 1: Figs. S17–S19) of the two AKI model mice after treatment for 14 days further demonstrated the remarkable therapeutic capacity of PB NZs to prevent AKI.

### Biomarker detection following treatment of mice with AKI

**Fig. 6:**
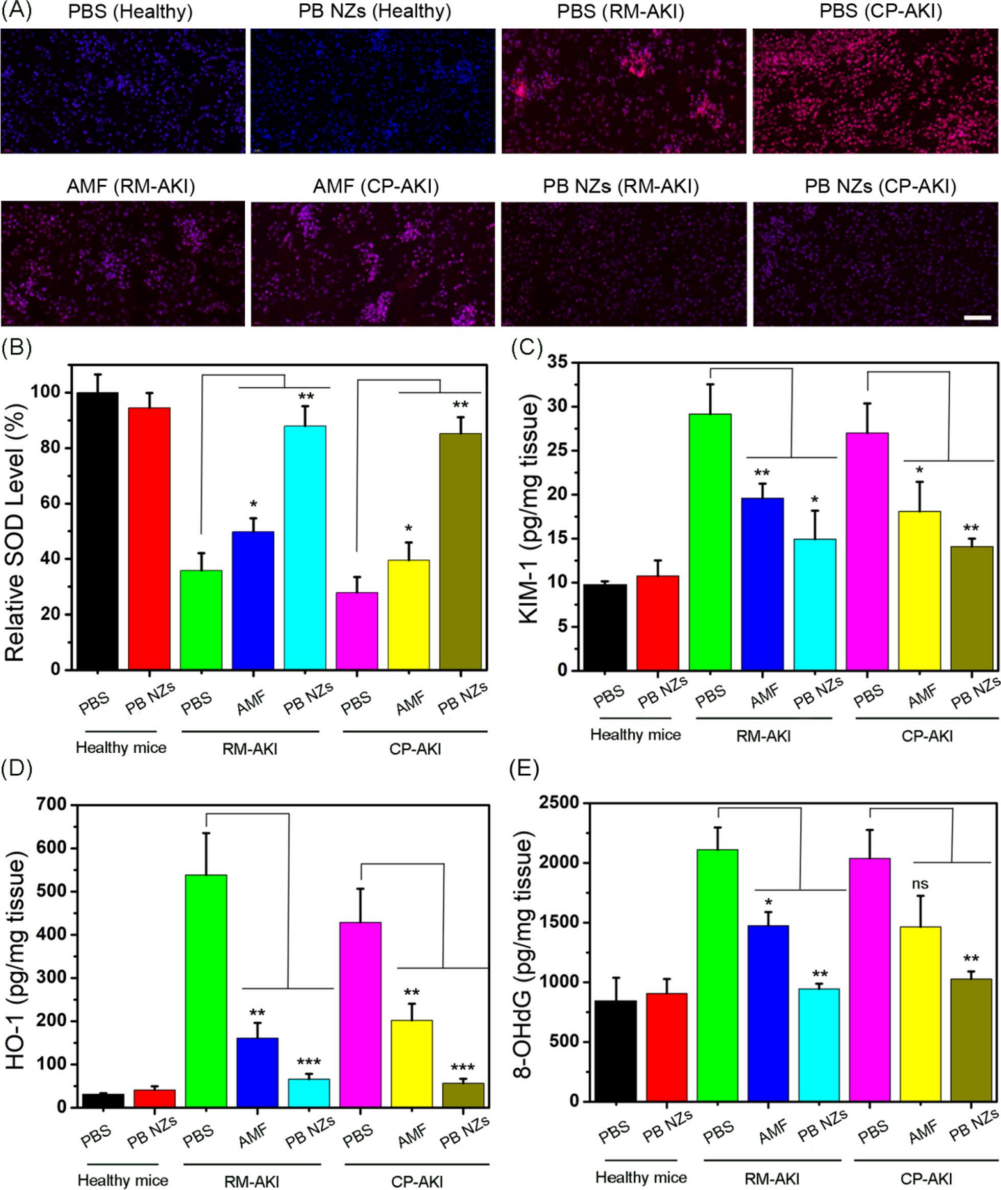
**A** DAPI and DHE staining of renal tissues collected from each group. The scale bar is 50 µm. **B** Relative SOD level in kidney homogenates after various treatments. Analysis of the biomarkers **C** KIM-1 and **D** HO-1 in kidney tissue homogenates collected from each group. **E** DNA damage measured in kidney tissue homogenates collected from each group. Data represent the mean ± SD (*n* = 3). *ns* non-significant, *P < 0.05, **P < 0.01, and ***P < 0.001 versus the RM-AKI or CP-AKI PBS-treated groups, respectively

Renal tissues were stained with dihydroethidium (DHE) and subjected to fluorescence imaging to determine renal superoxide production. An obvious inhibition of renal ROS levels was recorded in AKI mice treated with PB NZs (Fig. 6A), which confirmed the robust antioxidant capability of PB NZs. Similarly, PB NZs also reduced the level of RNS in both AKI models (Additional file 1: Fig. S20), offering an efficient protection to kidney cells. To directly observe the in vivo therapeutic effects of PB NZs, kidney tissues were homogenized to detec renal biomarkers. The level of SOD, a critical defense for neutralizing superoxide, was first measured in kidney homogenates [71]. Reduced SOD level was observed in both PBS and AMF treated groups, similar to what was seen in the healthy mice treated with PB NZs (Fig. 6B), suggesting that PB NZs could serve as antioxidants to scavenge RONS and protect renal cells. KIM-1 and HO-1 are key biomarkers of kidney injury [72, 73]. In two AKI mouse models treated with PB NZs, the expression of KIM-1 and HO-1 were significantly decreased to normal levels (Fig. 6C, D). We also investigated the ability of PB NZs to inhibit DNA damage and lipid peroxidation. Compared with the two AKI mouse models treated with PBS or AMF, the 8-hydroxy-2′-deoxyguanosine (8-OHdG) content, a quantitative biomarker of DNA damage, was significantly decreased in the kidneys of mice treated with PB NZs (Fig. 6E). Similarly, an increased lipid oxidation level was observed in the kidneys of PBS-treated mice while, similar to healthy mice, mice treated with PB NZs showed a remarkable decrease in their levels of lipid oxidation (Additional file 1: Fig. S21). These results indicated the good in vivo therapeutic efficiency of PB NZs in the treatment of AKI.

### In vivo toxicity of PB NZs

To assess the in vivo toxicity of PB NZs, blood and the major organs were collected from the mice after one month following *i.v.* injection of PB NZs. Compared with the organs and tissues in control groups, there was no obvious tissue damage or inflammatory lesions in the renal section of renal tubules, collecting tubules, glomeruli, ureters, or in the major organs (heart, liver, spleen, and lungs) of the experimental group, as shown in Additional file 1: Figs. S22, S23. Additionally, treatment with PB NZs did not alter the blood biochemistry of the mice as different biochemical indicators such as ALT, AST, BUN, and CRE were within normal range (Additional file 1: Fig. S24). Furthermore, no particular body-weight changes were noticed in either PBS- or PB NZs-treated mice (Additional file 1: Fig. S25). Collectively, these results verified an excellent biological safety of ultrasmall PB NZs and highlighted their potential applications in AKI theranostics.

## Conclusion

In summary, a multifunctional ultrasmall NZs-based on PB was successfully fabricated, which holds great promise to achieve dual-modal MR/PA imaging-guided AKI treatment. Using CS as a template, ultrasmall PB NZs were obtained by a simple solution reaction. The as-prepared PB NZs exhibited efficient cellular protection from H_2_O_2_-induced damage in vitro, because of their excellent multienzyme mimetic capability of removing excessive RONS. By virtue of their ultrasmall size, PB NZs present negligible systemic toxicity in vivo and effectively accumulate in the kidneys of AKI mice, as revealed by both MR and PA imaging. Impressively, these ultrasmall NZs, with their good biocompatibility and high level of enrichment in the kidneys, showed better remission results and therapeutic effects for AKI treatment in vivo than small molecule drugs, as demonstrated by serum and biomarker testing, histological staining, and mouse survival study. Our work highlighted the potential of ultrasmall PB NZs as a promising nanozyme platform for alleviating the symptoms of AKI, which is applicable for the clinical theranotics of AKI and other RONS-related diseases in the future.

## Supplementary Information

**Additional file 1.** Additional information includes part of material and methods, additional figures and table.

## Data Availability

All data used to generate these results is available in the main text and supporting information.

## Abbreviations

AKI: Acute kidney injury
RONS: Reactive oxygen/nitrogen species
AMF: Amifostine
ROS: Reactive oxygen species
NPs: Nanoparticles
NZs: Nanozymes
FDA: Food and Drug Administration
PB: Prussian blue
PB NZs: PB nanozymes
CAT: Catalase
H_2_O_2_: Hydrogen peroxide
PA: Photoacoustic
MR: Magnetic resonance
CS: Chitosan
POD: Peroxidase
SOD: Superoxide dismutase
·OH: Hydroxyl radical
O_2_^**.**^^−^: Superoxide anion
NO: Nitric oxide
ONOO^−^: Peroxynitrite
PBS: Phosphate buffered saline
ICP-MS: Inductively coupled plasma mass spectrometry
HEK293T: Human embryonic kidney 293T
MTT: 3-(4,5-Dimethyl-2-thiazolyl)-2,5-diphenyl-2*H*-tetrazolium bromide
OD: Optical density
DCF: 2′,7′-Dichlorodihydrofluorescein diacetate
DAF-FM DA: 3-Amino,4-aminomethyl-2′,7′-difluorescein diacetate
FBS: Fetal bovine serum
DMEM: Dulbecco’s modified Eagle’s medium
BUN: Blood urea nitrogen
CRE: Creatinine
H&E: Hematoxylin and eosin
HO-1: Heme oxygenase-1
KIM-1: Kidney injury molecule-1
ELISA: Enzyme linked immunosorbent assay
TBARS: Thiobarbituric acid-reactive substances
AST: Aspartate aminotransferase
ALT: Alanine aminotransferase
RM: Rhabdomyolysis-induced
CP: Cisplatin-induced
AFM: Atomic force microscopy
DLS: Dynamic light scattering
TEM: Transmission electron microscopy
XRD: X-ray powder diffraction
XPS: X-ray photoelectron spectroscopy
FTIR: Fourier-transform infrared
TGA: Thermogravimetric analysis
Ru(dpp)_3_Cl_2_: Tris (4,7-diphenyl-1,10-phenanthroline) ruthenium(II) dichloride
TMB: Tetramethyl benzidine
DMPO: 5,5-Dimethyl-1-pyrroline *N*-oxide
·ABTS: 2,2′-Azino-bis(3-ethylbenzothiazoline 6-sulfonate radical
RNS: Reactive nitrogen species
DPPH: 2,2-Di-(4-*tert*-octylphenyl)-1-picrylhydrazyl
*i.m.*: Intramuscular
*i.v.*: Intravenous
*p.i.*: Post-injection
NIR: Near-infrared
*i.p.*: Intraperitoneal
TUNEL: Terminal-deoxynucleotidyl transferase mediated nick end labeling
DHE: Dihydroethidium
