# Supplementary material for: Prussian blue-based theranostics for ameliorating acute kidney injury
Source: J Nanobiotechnology. 2021 Sep 6;19:266. doi: 10.1186/s12951-021-01006-z (PMC8419910; doi:10.1186/s12951-021-01006-z)
Supplement: Supplementary file 1 — Additional file 1. Additional information includes part of material and methods, additional figures and table. [file 12951_2021_1006_MOESM1_ESM.docx]

Additional file 1

Prussian Blue-Based Theranostics for Ameliorating Acute Kidney Injury

Dong-Yang Zhang^1,2,†^, Hengke Liu^1,†^, Kathy S. Zhu^1,3^, Ting He^1^, Muhammad Rizwan Younis^1,2^, Chen Yang^1,2^, Shan Lei^1,2^, Jiayingzi Wu^1^, Jing Lin^1^, Junle Qu^2^, and Peng Huang^1^*

^1^Marshall Laboratory of Biomedical Engineering, International Cancer Center, Laboratory of Evolutionary Theranostics (LET), School of Biomedical Engineering, Shenzhen University Health Science Center, Shenzhen 518060, China

^2^Key Laboratory of Optoelectronic Devices and Systems of Ministry of Education and Guangdong Province, College of Optoelectronic Engineering, Shenzhen University, Shenzhen 518060, China

^3^National Clinical Research Center for Oral Diseases, National Engineering Laboratory for Digital and Material Technology of Stomatology, Beijing Key Laboratory of Oral Digital Medicine, Peking University School and Hospital of Stomatology, Beijing 100081, China

Corresponding authors Email: [peng.huang@szu.edu.cn](mailto:peng.huang@szu.edu.cn).

^†^These authors contributed equally: Dong-Yang Zhang and Hengke Liu.

**Material and methods**

**Materials**

Chitosan (CS), glycerinum, and 2,2′-azino-bis(3-ethylbenzothiazoline 6-sulfonate (ABTS) were obtained from Sigma-Aldrich (USA). [Potassium](E:/%E6%99%AE%E9%80%9A%E8%BD%AF%E4%BB%B6/%E6%9C%89%E9%81%93%E8%AF%8D%E5%85%B8/Dict/8.8.1.0/resultui/html/index.html#/javascript:;) [ferricyanide](E:/%E6%99%AE%E9%80%9A%E8%BD%AF%E4%BB%B6/%E6%9C%89%E9%81%93%E8%AF%8D%E5%85%B8/Dict/8.8.1.0/resultui/html/index.html#/javascript:;), ferrous chloride hydrate, catalase, 3,3',5,5'-Tetramethylbenzidine dihydrochloride (TMB), potassium persulfate, 2,2-di-(4-tert-octylphenyl)-1-picrylhydrazyl (DPPH) and 5,5-dimethyl-1-pyrroline N-oxide (DMPO) were purchased from Aladdin Reagent Co., Ltd. (China). Cisplatin was purchased from J&K Scientific Co. Ltd. (China). Tris (4,7-diphenyl-1,10-phenanthroline) ruthenium (II) dichloride (Ru(dpp)_3_Cl_2_) was bought from Meryer Chemical Technology Co., Ltd. (China). Deionized water, purified by a Milli-Q water purification system (Millipore, USA) to a minimum resistivity of 18.2 MΩ/cm, was used in all experiments.

**Measurement of CAT-like activity**

The assays to determine the catalase-like activity of PB NZs were conducted at 25 ℃ and measured the O_2_ generated using a specific oxygen electrode on a Multi-Parameter Analyzer (DZS-708, Cany, China). A 0.2 ml volume of PB NZs solution (at concentrations of 0, 4, or 8 mg/mL) was added to 3.8 ml of 3% H_2_O_2_ solution, which had been diluted in PBS (pH 7.4). The solubility of the O_2_ generated (unit: mg/L) was measured every 1 min for 30 min. In order to verify the generation of O_2_ bubbles, different concentrations of PB NZs solution were mixed with 3wt% H_2_O_2_ diluted with PBS in 4 mL centrifuge tubes at 37 °C, allowed to react for 20 min, and then photographed.

The production of O_2_ was further demonstrated by experiments with ruthenium complexes [(Ru(dpp)_3_)]Cl_2_, which are used as commercial oxygen sensing probes. PBS (150 μL), 3.0 wt% H_2_O_2_ (150 μL in PBS), PB NZs solution (150 μL in PBS) or PB NZs solution + 3.0 wt% H_2_O_2_ (150 μL in PBS) was added to a 96-well plate with [(Ru(dpp)_3_)]Cl_2_ (10 µg/mL, 10 µL) and incubated at 37 ℃ for 30 min. Then, a microplate reader was used to record the fluorescence in each well. Next, 0, 0.75, 1.5, 3, or 6 wt% H_2_O_2_ (final concentration) and 200 µg/mL PB NZs in 150 μL PBS (pH 7.4) were added to the 96-well plate, mixed with [(Ru(dpp)_3_)]Cl_2_ (10 µg/mL, 10 µL), and incubated at 37 °C for 30 min. The phosphorescence intensity of each well was determined. The emission and excitation wavelengths used were 620 nm and 488 nm, respectively.

**Measurement of POD-like activity**

Steady-state kinetic assays of POD-like activity were carried out at room temperature in 3 ml PBS (pH 7.4) with PB NZs (20 μg/mL) in the presence of TMB and H_2_O_2_. The kinetic analysis of PB NZs with H_2_O_2_ as a substrate was performed using different concentrations of TMB but at a fixed concentration of H_2_O_2_ in PBS (pH 7.4), and vice versa. The absorbance of oxidized TMB products at 650 nm was monitored in time scan mode using a Cary 60 UV-vis-NIR spectrophotometer (Agilent, USA). The kinetic parameters were determined by fitting data to the Michaelis-Menten equation: *V*=*V*_max_*C*/(*K*_m_+*C*), where *V* is the initial velocity, *V*_max_ is the maximum rate of conversion, *C* is the substrate concentration, and *K*_m_ is the Michaelis constant.

**Scavenging** •**OH and O_2_**^•^**^-^ of PB NZs**

The •OH and O_2_^•-^ scavenging ability of PB NZs was evaluated using a •OH antioxidant capacity assay kit (Cell Biolabs, Inc., USA) and a SOD assay kit (Sigma-Aldrich, USA), respectively. Experiments were carried out according to the manufacturers’ protocols.

The •OH and O_2_^•-^ scavenging ability of PB NZs was evaluated by electron spin resonance (ESR) assay. 50 μM FeCl_2_, 5 mM H_2_O_2_, 100 mM DMPO, and 100 μg/mL of PB NZs were mixed in PBS (pH 7.4). Then, ESR signal was measured immediately. The ESR signals of sample containing 10 mM KO_2_ in DMSO, 100 μg/mL of PB NZs, and 100 mM DMPO were recorded.

**Scavenging** •**ABTS of PB NZs**

An ABTS radical cation decolorization assay was used to measure the free radical scavenging activity of PB NZs. •ABTS were produced by mixing ABTS (7 mM) and potassium persulfate (2.45 mM) in deionized water for 24 h. Subsequently, the UV-vis absorbance values at 734 nm of 12.5, 25, and 50 μg/mL of PB NZs (A_PB_), pure •ABTS solution (A_B_), and •ABTS solution with PB NZs (A_P_) were measured. The inhibition ratio of •ABTS was calculated using the following formula: [(A_B_ - A_P_ - A_PB_)/A_B_] × 100.

**Scavenging DPPH radicals of PB NZs**

DPPH radicals were employed to evaluate the RNS scavenging activity of PB NZs. Different concentrations of PB NZs were mixed with DPPH (40 μM) for 12 h and the absorption spectra were recorded. In addition, the reaction was started with the addition of PB NZs (200 μg/mL) and the absorption spectra were recorded for 50 min using a UV-Vis spectrophotometer.

**Cell lines and culture conditions**

Human embryonic kidney 293T (HEK293T) cells were obtained from the OBiO Technology Corp., Ltd (Shanghai, China). Cells were cultured in Dulbecco’s modified Eagle’s medium (DMEM) (Gibco BRL) supplemented with 1% penicillin/streptomycin and 10% fetal bovine serum at 37 °C under 5% CO_2_.

**Cytotoxicity and hemolysis assays**

The HEK293T cells were seeded in 96-well plates and cultured for 24 h at 37 ℃. Then, the cells were treated with PB NZs; the concentration varied from 0 to 200 µg/mL. After 20 or 44 h, 20 μl MTT (5 mg/mL) solution was added to each well. The plates were incubated in the dark at 37 ℃ for an additional 4 h. The medium was carefully removed and 150 µL dimethylsulfoxide was added to each well. The optical density (OD) at 570 nm was measured using a microplate reader. Cells treated under identical conditions in the dark were kept as a control group. Cell viability was calculated based on the following formula: viable cells (%) = (OD of treated sample/OD of untreated sample) × 100%.

Fresh blood was drawn from healthy mice. The blood samples were centrifuged at 3000 rpm and washed several times with PBS. To study the hemolysis caused by PB NZs, 0.3 mL of the blood dispersion was mixed with 1.2 mL of water, PBS, and PB NZs at various concentrations (0.25, 0.5, 1, 2, 4, and 8 mg/mL). Then, the mixtures were incubated for 4 h at room temperature. After the mixtures were centrifuged at 4000 rpm for 5 min, the absorbance of the supernatants at 541 nm was recorded using a microplate reader. The hemolysis rate of RBCs was determined according to the formula: Hemolysis (%) = (A_sample_ - A_PB NZs_ - A_PBS_)/(A_water_ - A_PBS_) × 100%, where A_sample_, A_PB NZs_, A_PBS_, and A_water_ are the 541 nm absorbance of the sample groups, PB NZs NP solutions, the PBS group, and the water group, respectively.

**RONS scavenging by PB NZs in cells**

The HEK293T cells were seeded into 35 mm dishes and treated with or without 200 μg/mL PB NZs for 4 h. Then, DCF (Sigma-Aldrich, USA), [Mito-Tracker Green](https://www.beyotime.com/product/C1048.htm) (Beyotime Biotechnology, China), DAF-FM DA (Beyotime Biotechnology, China), or DAX-J2^TM^ PON Green ([AAT Bioquest, Fluorimetric Intracellular Peroxynitrite Assay Kit](http://dzyh.szu.edu.cn/lab/orders/product/80040011089274/1), USA) in FBS-free DMEM medium was added to the cells after the treatment of PB NZs with 2 mM H_2_O_2_. After 30 min incubation at 37 ℃, the cells were further washed and analyzed under confocal laser microscopy.

PB NZs dispersions at different concentrations were incubated with HEK293T cells in confocal dishes for 4 h at 37 ℃. Then, the medium was replaced with 2 mM H_2_O_2_ in PBS and incubated for 15 min at 37 ℃. The cells were washed twice with PBS and incubated with DCF, DAF-FM DA, or DAX-J2^TM^ PON Green in FBS-free DMEM medium for 15 min at 37 ℃ in the dark. Finally, fluorescence in HEK293T cells was detected by flow cytometry and quantified by the software FlowJo X 10.0.7.

Using 96-well culture plates, the HEK293T cells were seeded at 10^4^ cells per well and then incubated for 24 h with 5% CO_2_ at 37 ℃. The PB NZs dispersions with different concentrations (0-200 μg/mL) were incubated with cells for 4 h at 37 ℃. After treatment with H_2_O_2_ (500 μM) for 24 h at 37°C, the cellular viability was measured using an MTT assay.

Using 6-well culture plates, the HEK293T cells were seeded and incubated for 24 h. The PB NZs dispersions with different concentrations (0–200 μg/mL) were incubated with cells for 4 h. After treatment with H_2_O_2_ (500 μM) for 24 h at 37°C, cells stained with propidium iodide/Annexin V-FITC were detected by flow cytometry.


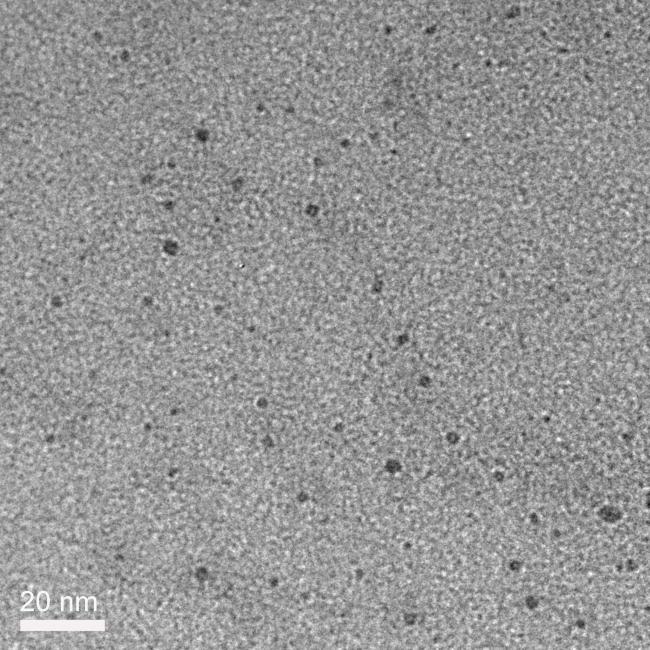


Figure S1. TEM image of PB NZs. The scale bar is 20 nm.


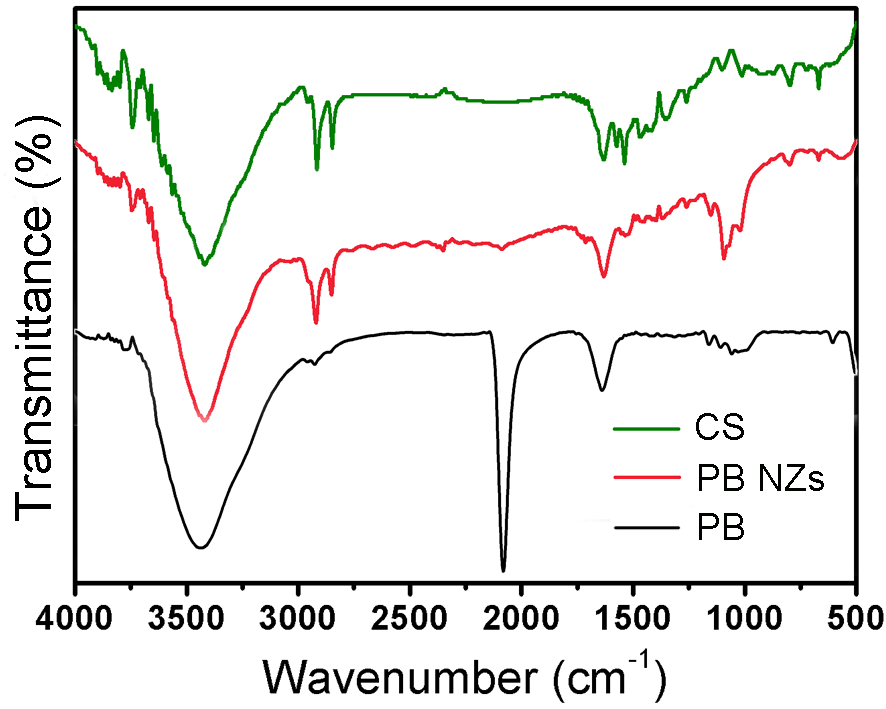


Figure S2. FTIR spectra of CS and PB NZs.


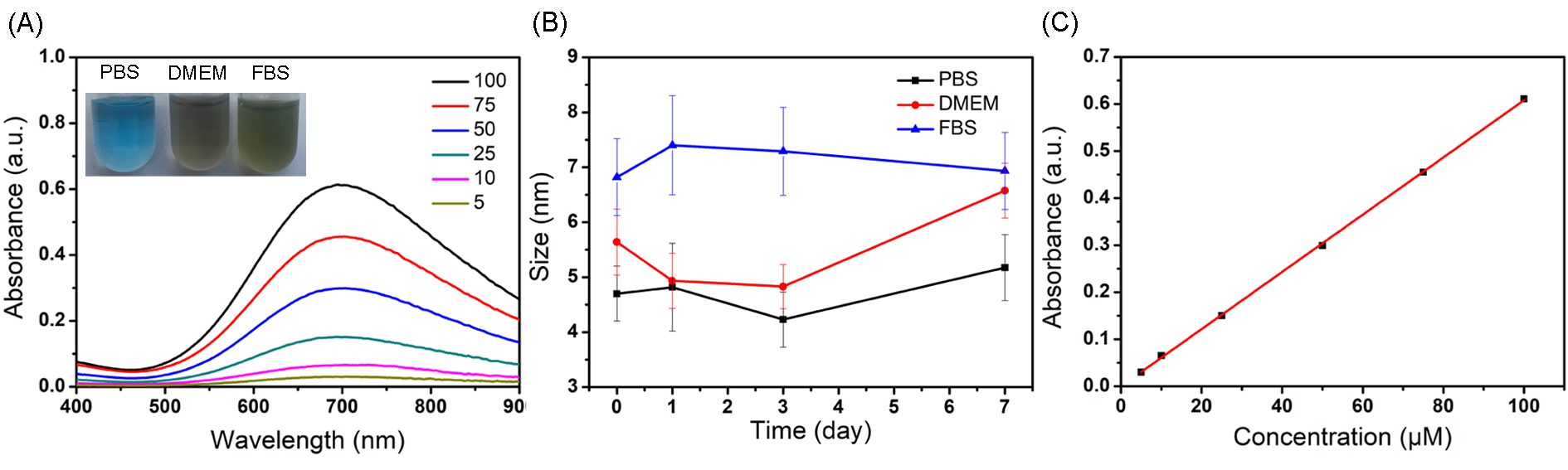


Figure S3. (A) Absorbance spectra of the PB NZs in aqueous solution at different concentrations. The unit is 100 ppm Fe and the inset image is the PB NZs solution in PBS, DMEM, and FBS. (B) Size distribution of PB NZs in PBS, DMEM, and FBS for 7 days. (C) Optical absorbance at 700 nm for aqueous suspensions of various concentrations of PB NZs with an optical path of 1 cm. The solid line is Beer’s Law fit for obtaining the molar extinction coefficient. Unit: μM.


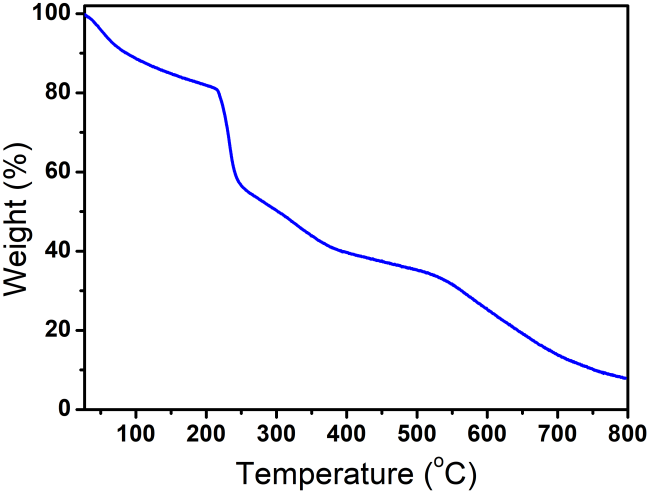


Figure. S4 TGA curve obtained from PB NZs.


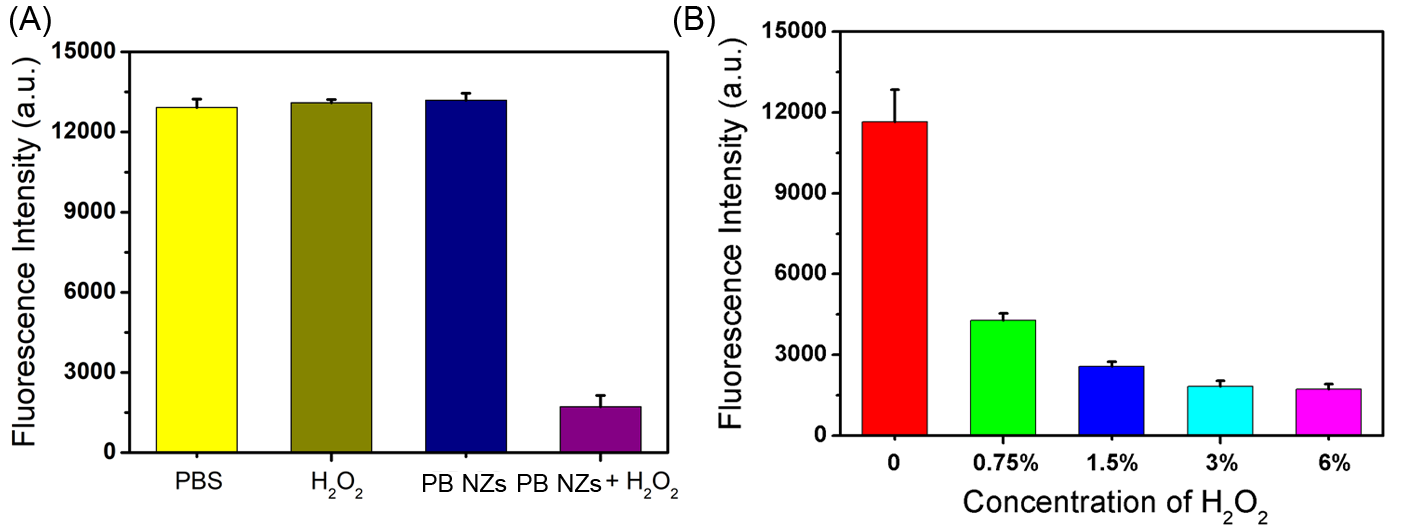


Figure S5. (A) Fluorescence intensity of [Ru(dpp)_3_]Cl_2_ added to mixtures containing PBS, H_2_O_2_, PB NZs, or PB NZs + H_2_O_2_. [PB NZs]: 200 μg/mL, [H_2_O_2_]: 3 wt%. (B) Fluorescence intensity of [Ru(ddp)_3_]Cl_2_-added to PB NZs (200 μg/mL) solution incubated with various concentrations of H_2_O_2_.


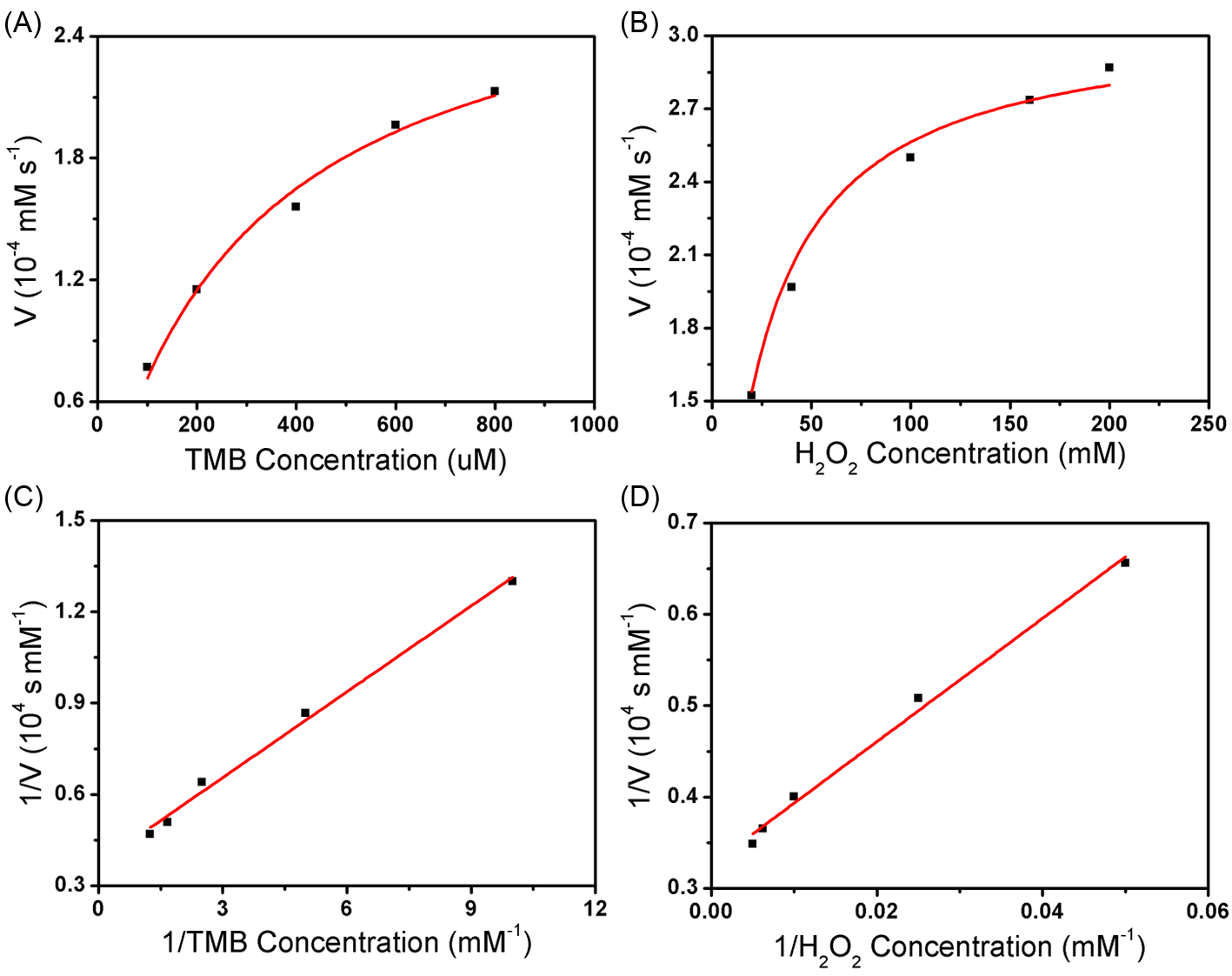


Figure S6. Steady-state kinetic assay of POD-like activity of PB NZs in PBS (pH 7.4, 10 mM). Kinetic analysis of PB NZs with TMB (A) and H_2_O_2_ (B) as substrates, respectively. (C, D) Double-reciprocal plots of PB NZs activity corresponding to (A, B).


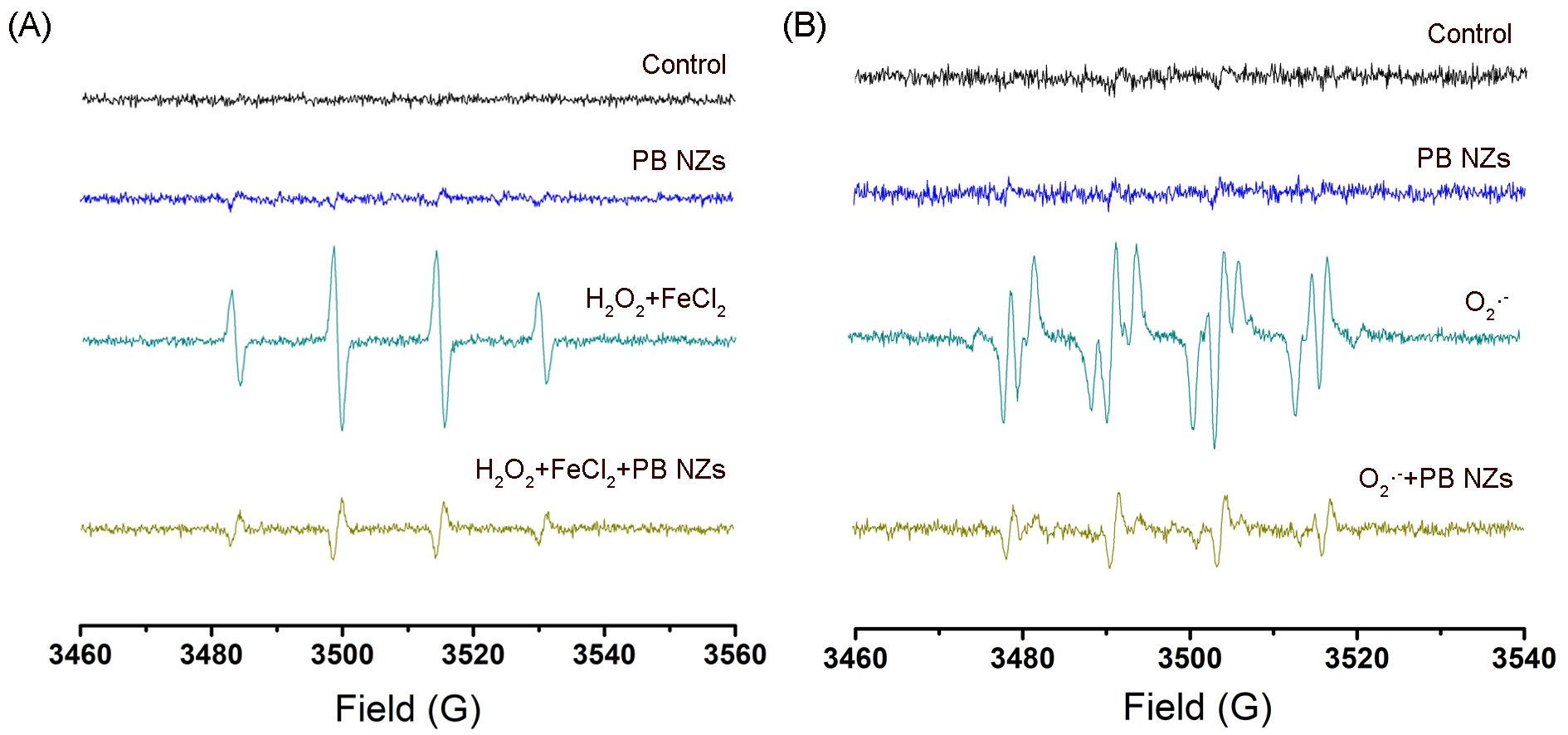


Figure S7. (A) ESR spectra of 5,5-dimethyl-1-pyrroline N-oxide (DMPO)-•OH adduct were observed during incubation of FeCl_2_ (20 μM), H_2_O_2_ (5 mM), and PB NZs (100 μg/mL). (B) ESR spectra of the DMPO-O_2_^•-^ adduct were observed during incubation of KO_2_ (2.5 mM) containing PB NZs (100 μg/mL) and DMPO (100 mM) in DMSO solution.


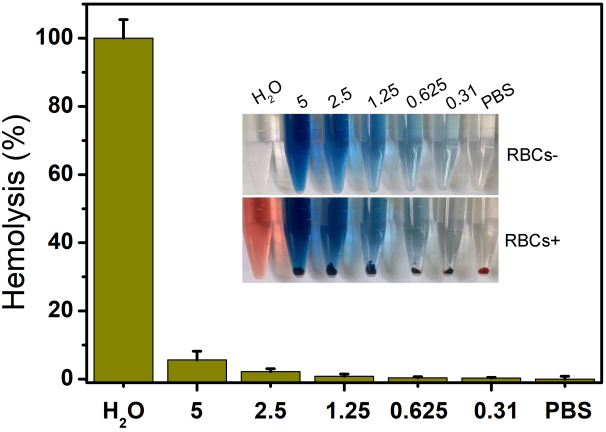


Figure S8. Hemolysis test of PB NZs in red blood cells (RBCs). The unit is mg/mL.


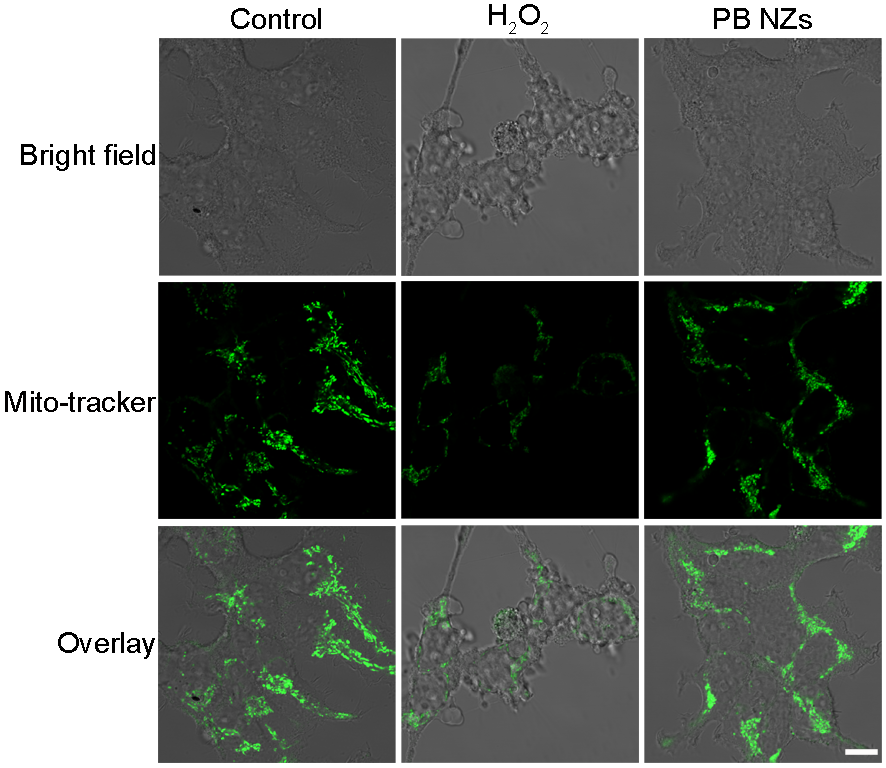


Figure S9. Representative confocal fluorescence microscopy images of mitochondrial staining of HEK293T cells with different treatments, as mentioned. (Scale bar: 5 μm.)


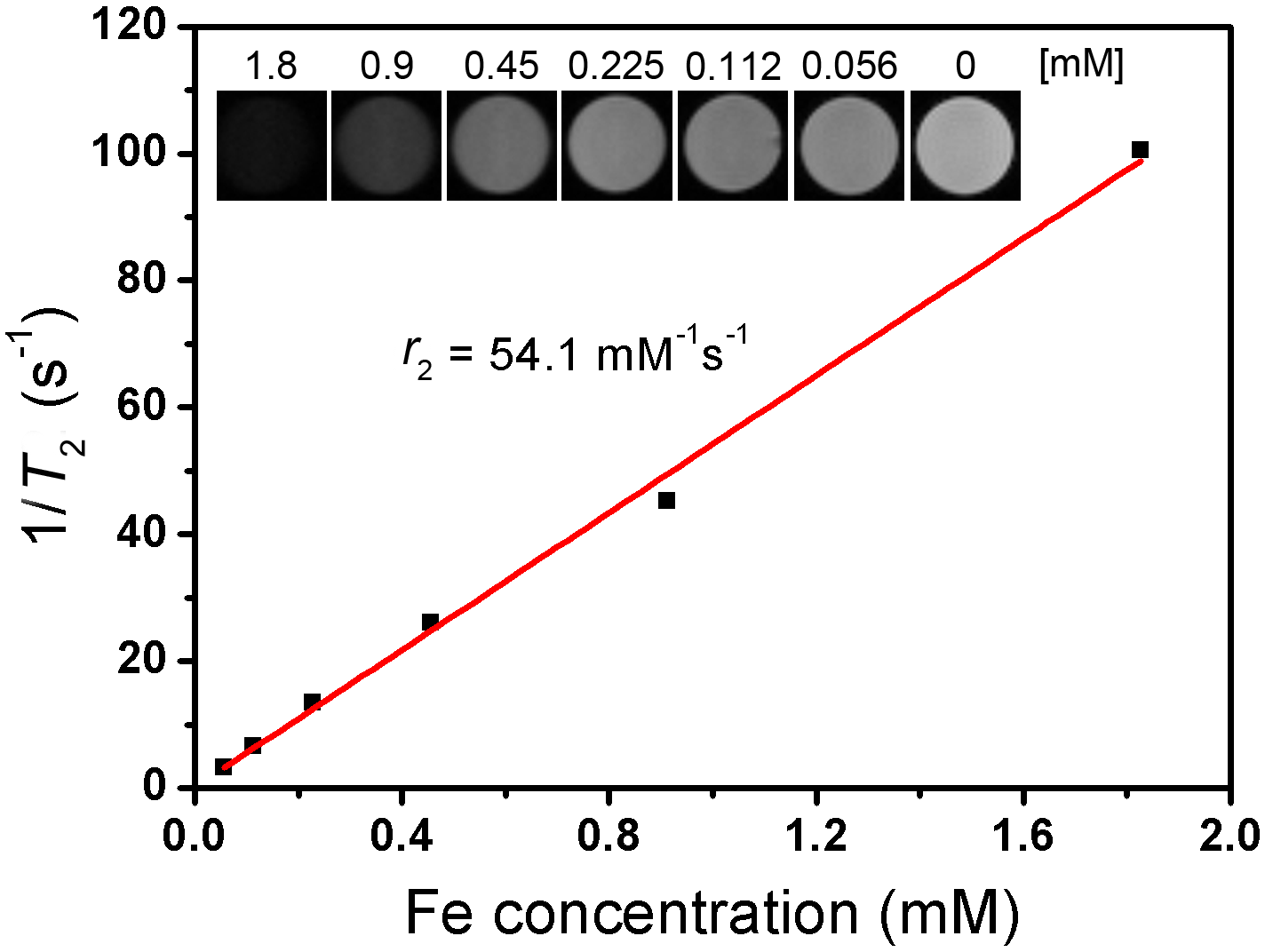


Figure S10. *T_2_*-weighted magnetic resonance images and *T_2_* relaxation rates of indicated concentrations of Fe from PB NZs.


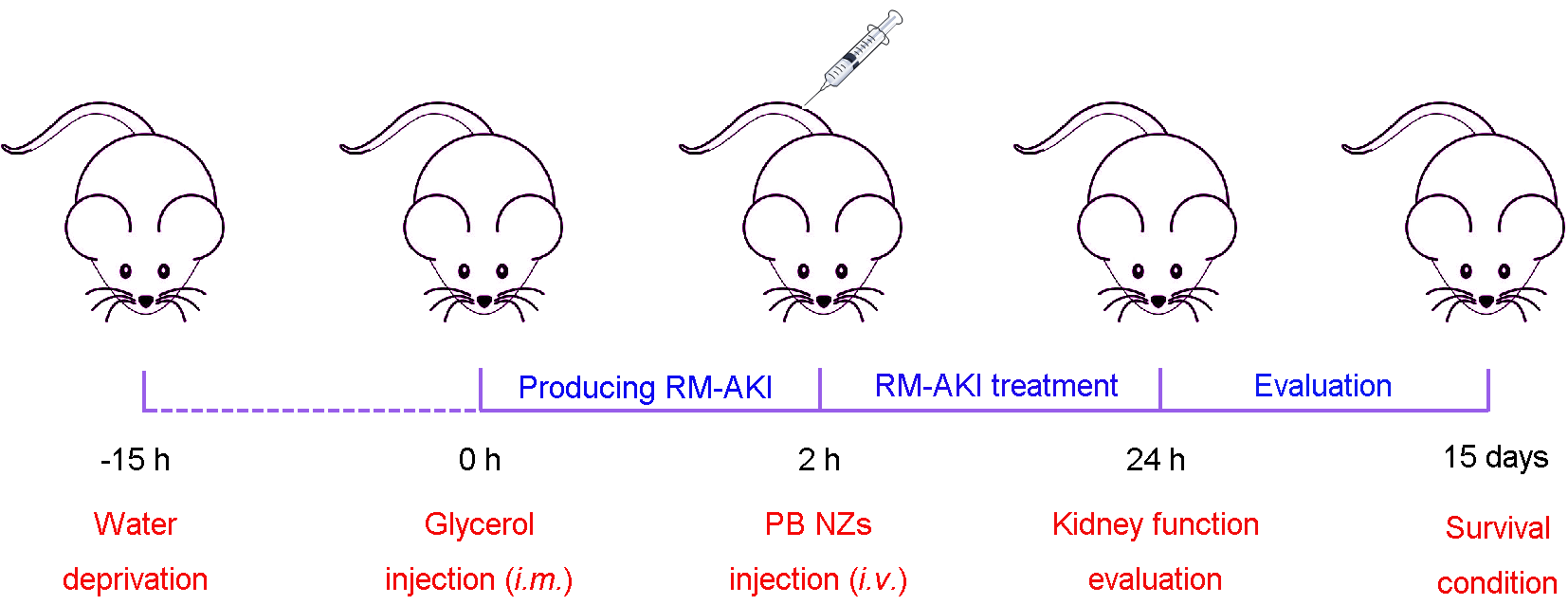


Figure S11. Preparation and treatment schedule for RM-AKI mice.


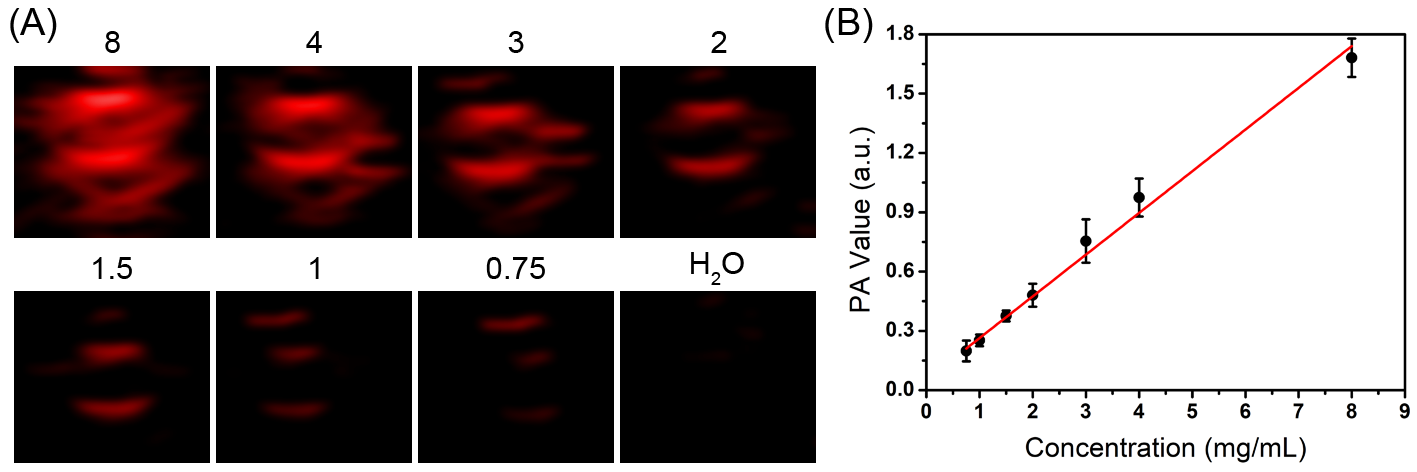


Figure S12. Photoacoustic (PA) images (A) and PA amplitudes (B) of PB NZs dispersions at various concentrations (unit: mg/mL) upon 700 nm laser irradiation.


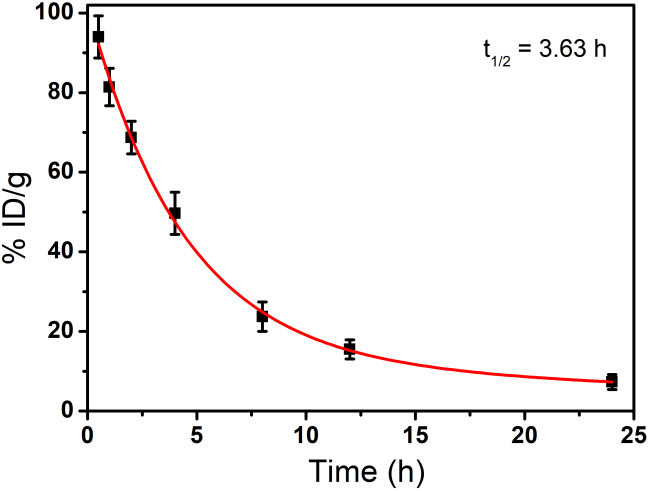


Figure S13. Blood circulation of PB NZs after intravenous injection in healthy mice (*n* = 3).


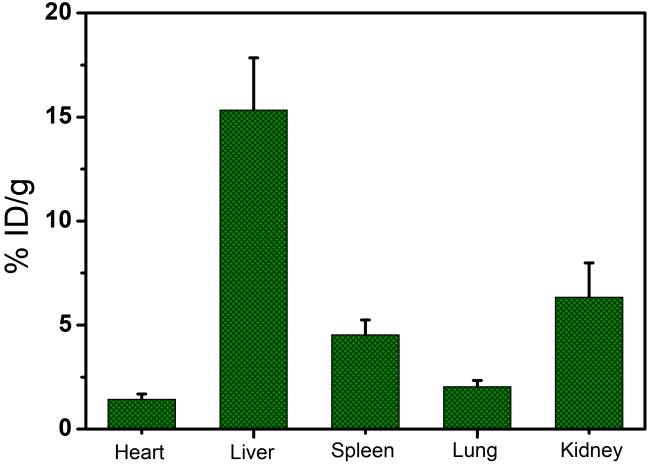


Figure S14. Biodistribution of PB NZs in the major organs at 24 h after *i.v.* injection. Data represent means ± S.D. (*n* = 3).


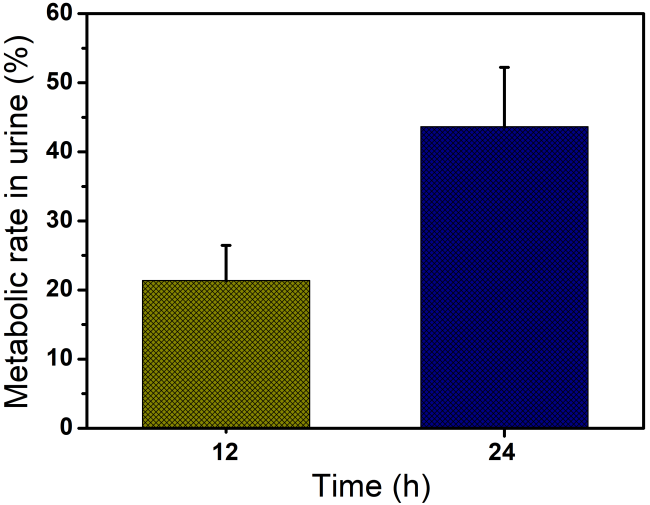


Figure S15. The percentage of Fe in urine at different time points (12 and 24 h) from the total intravenously injected PB NZs.


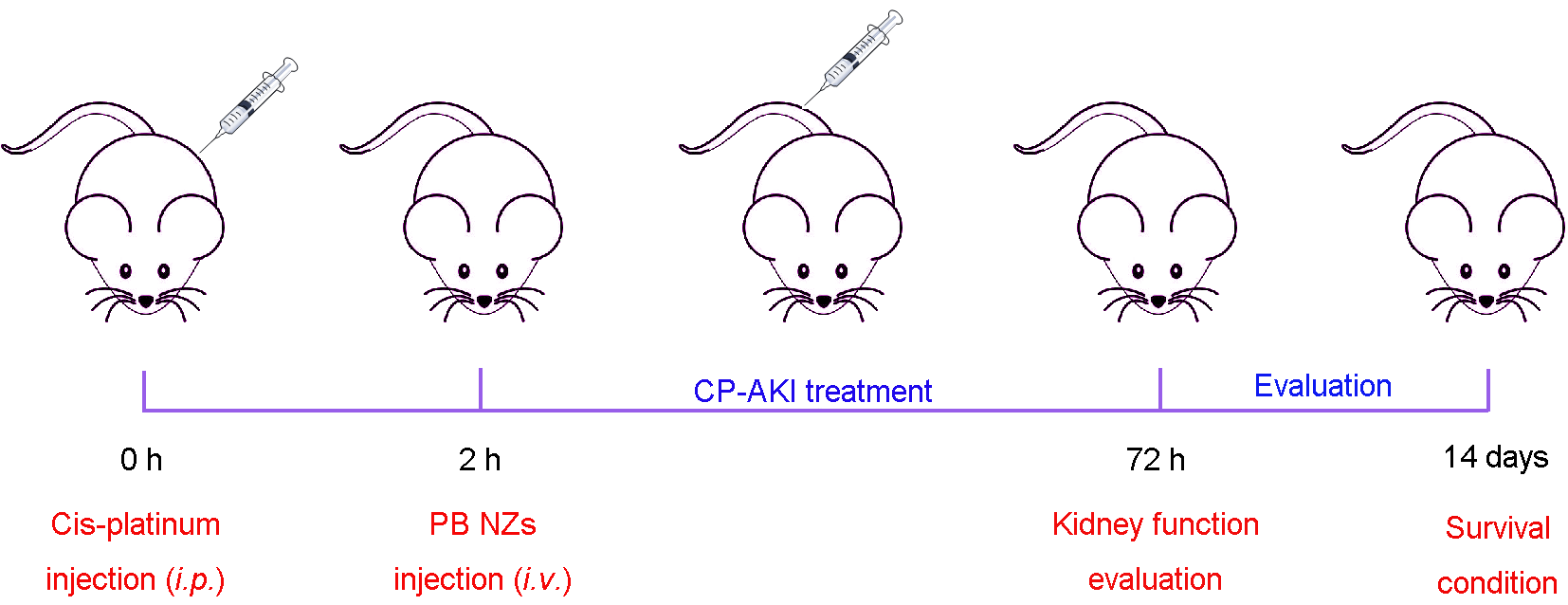


Figure S16. Preparation and treatment schedule for CP-AKI mice.


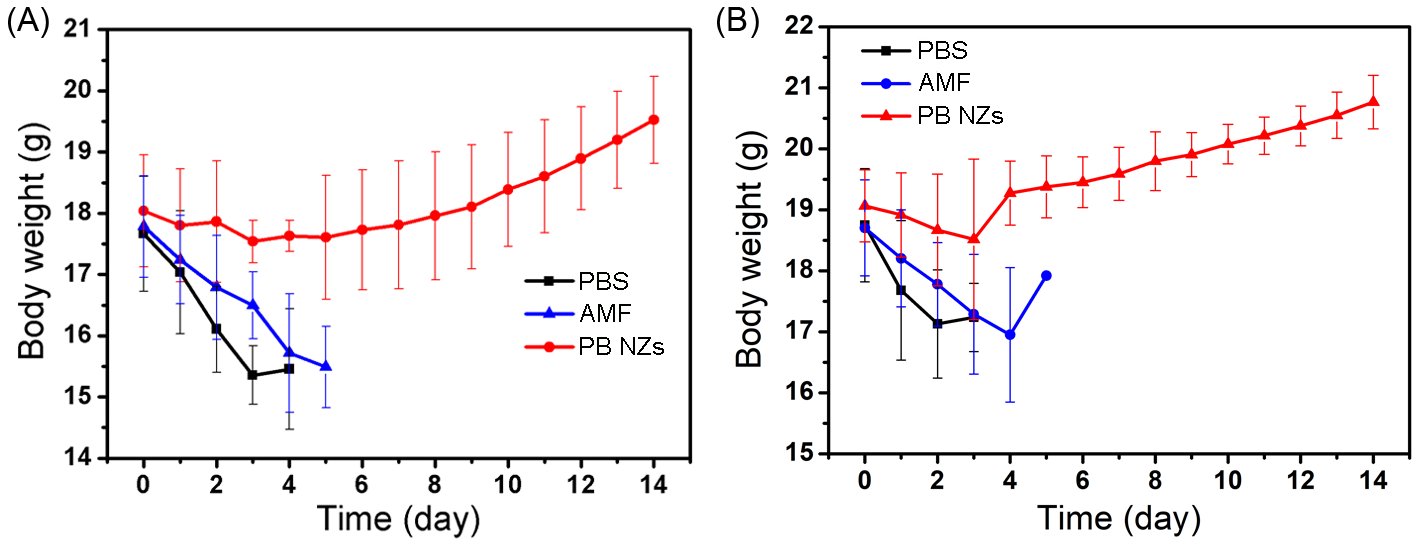


Figure S17. Changes in body weights of (A) RM-AKI and (B) CP-AKI mice over 14 days, following PBS, AMF, or PB NZs treatment.


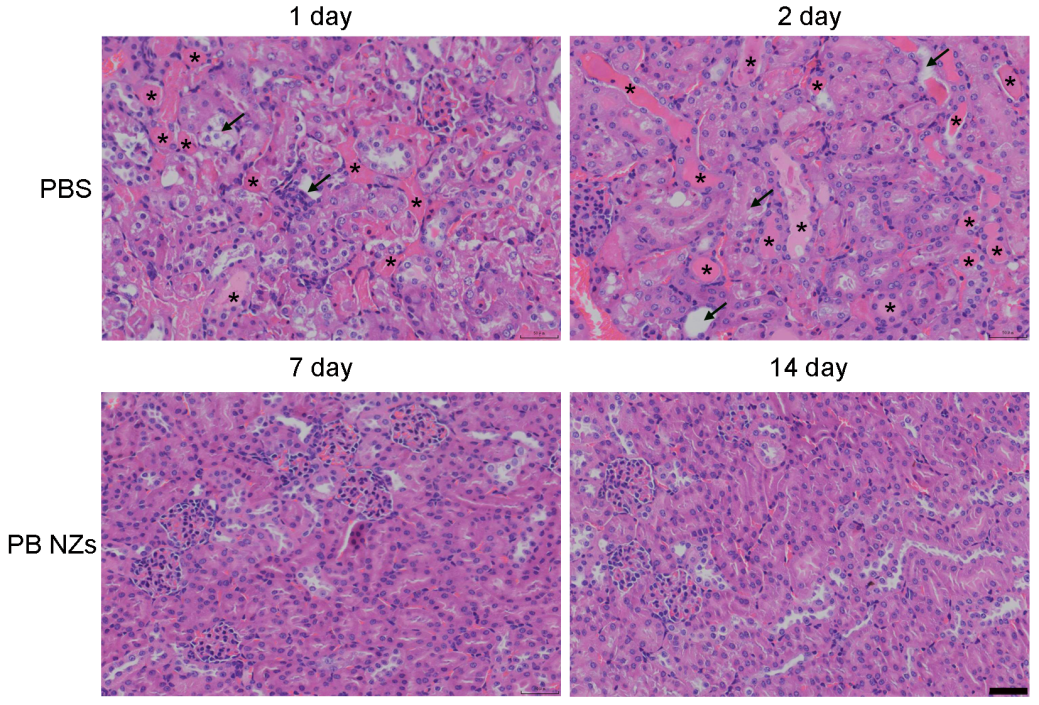


Figure S18. Treatment of RM-AKI. H&E stained images of kidney tissues harvested from PBS- or PB NZs-treated mice. (Scale bar: 50 μm.) Arrows indicate damaged tubules and asterisks indicate the formation of casts.


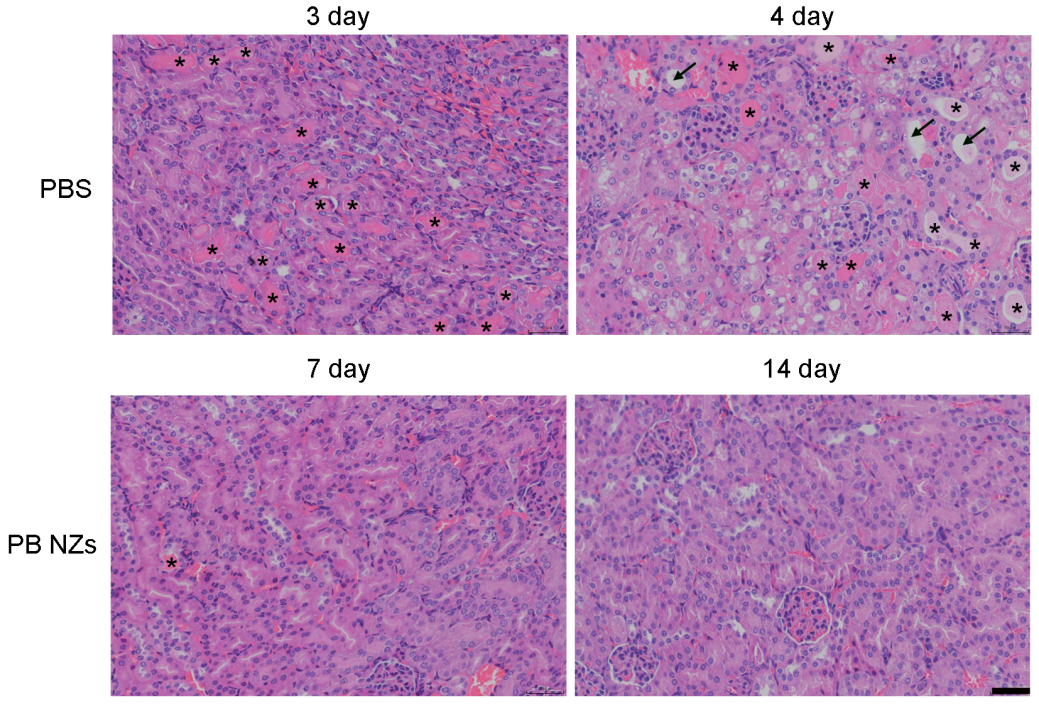


Figure S19. Treatment of CP-AKI. H&E stained images of renal tissues harvested from PBS- or PB NZs-treated mice. (Scale bar: 50 μm.) Arrows indicate damaged tubules and asterisks indicate the formation of casts.


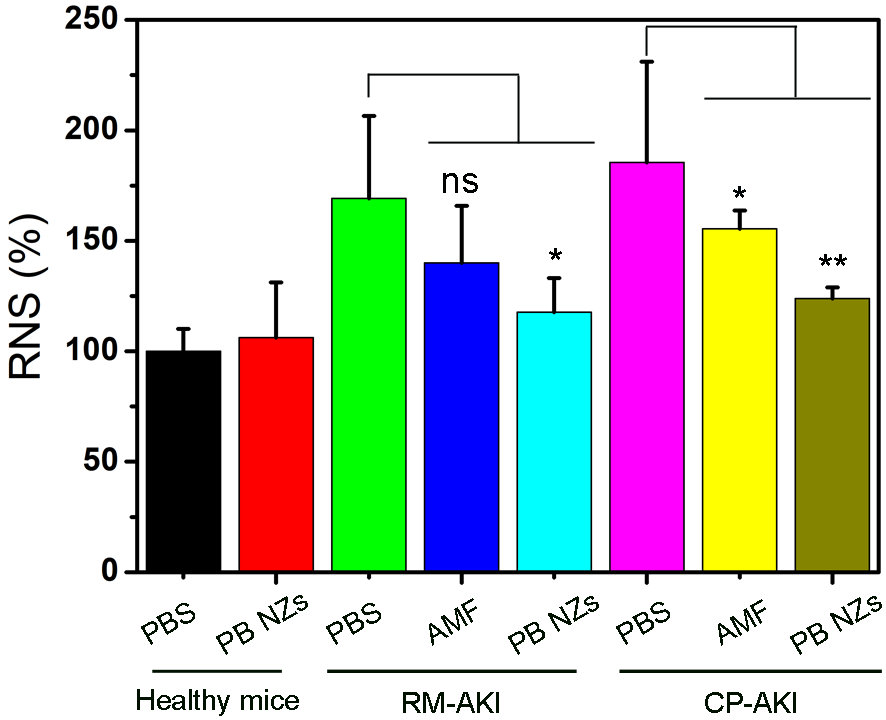


Figure S20. Determination of RNS in the kidneys of healthy (control), RM-AKI, and CP-AKI mice groups, (*n* = 3, mean ± S.D.).


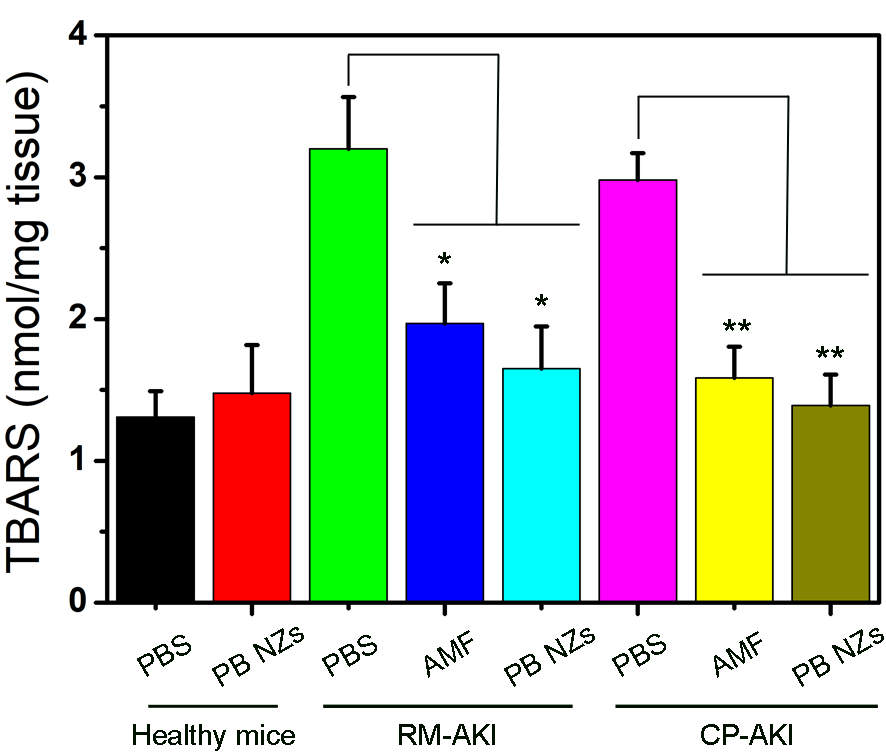


Figure S21. Determination of TBARS in the kidneys of mice from the control, RM-AKI, and CP-AKI groups, (*n* = 3, mean ± S.D.).


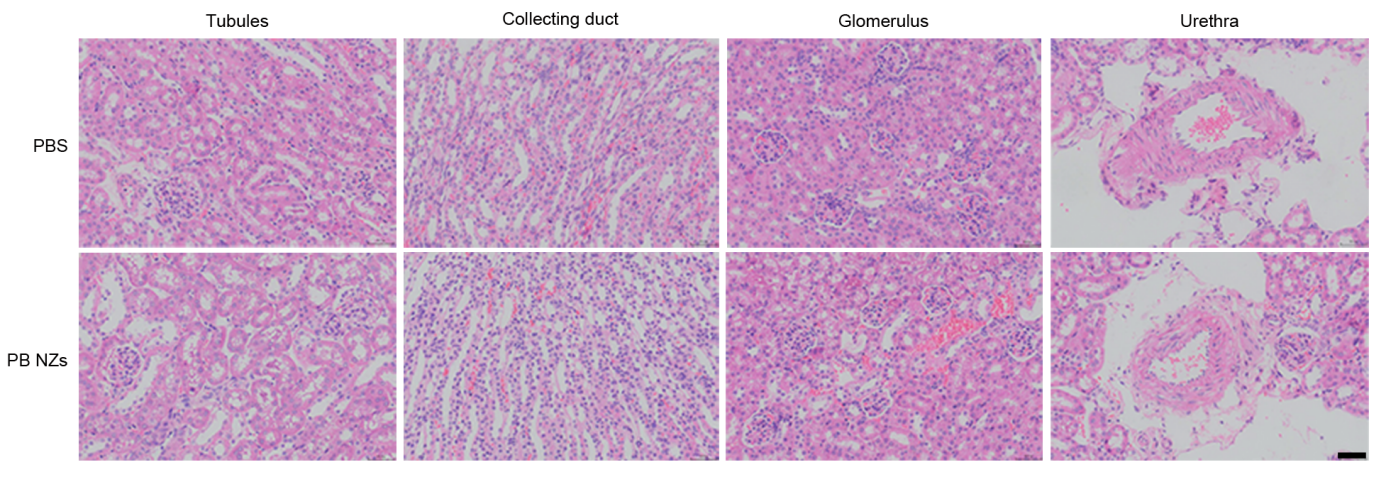


Figure S22. H&E stained images of the kidneys (tubules, collecting duct, glomerulus, and urethra). The scale bar is 100 µm.


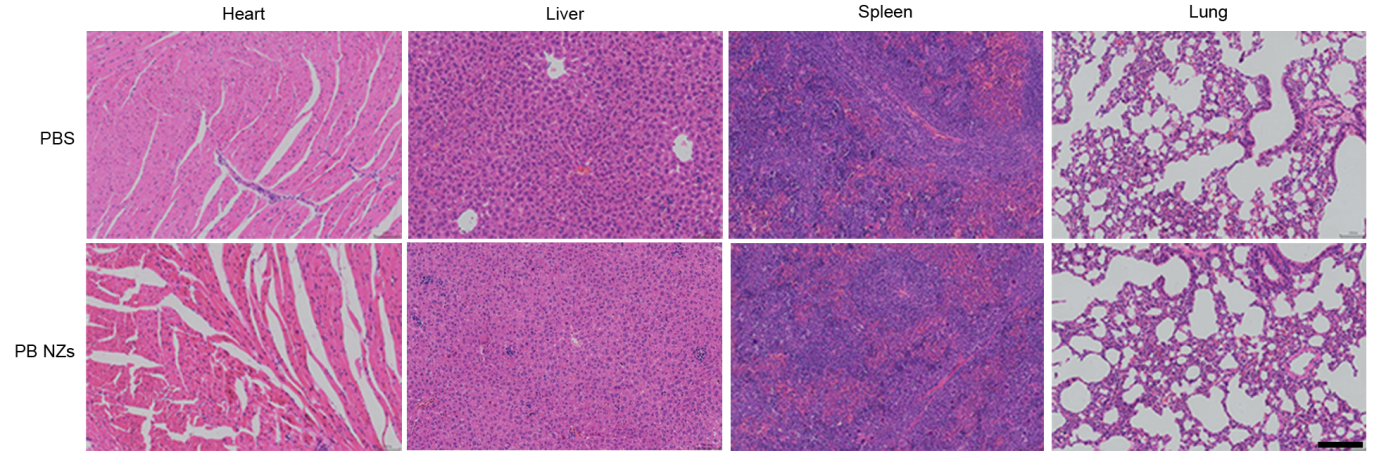


Figure S23. H&E stained images of primary organs (heart, liver, spleen, and lungs) following the indicated treatments. scale bar: 200 µm.


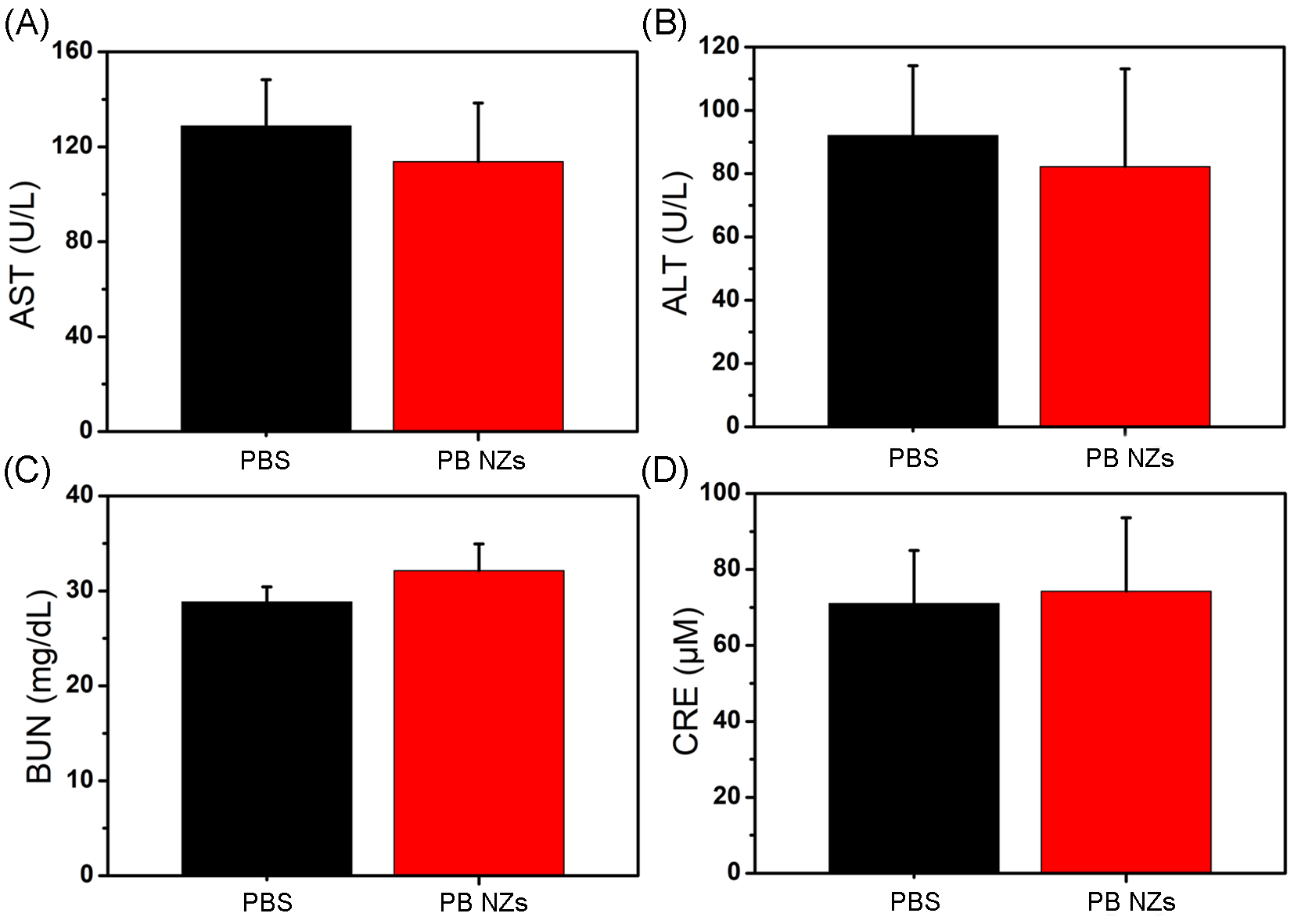


Figure S24. Blood biochemistry analysis (AST, ALT, BUN, and CRE) of healthy BALB/c mice after intravenous injection of PBS or PB NZs for 14 days.


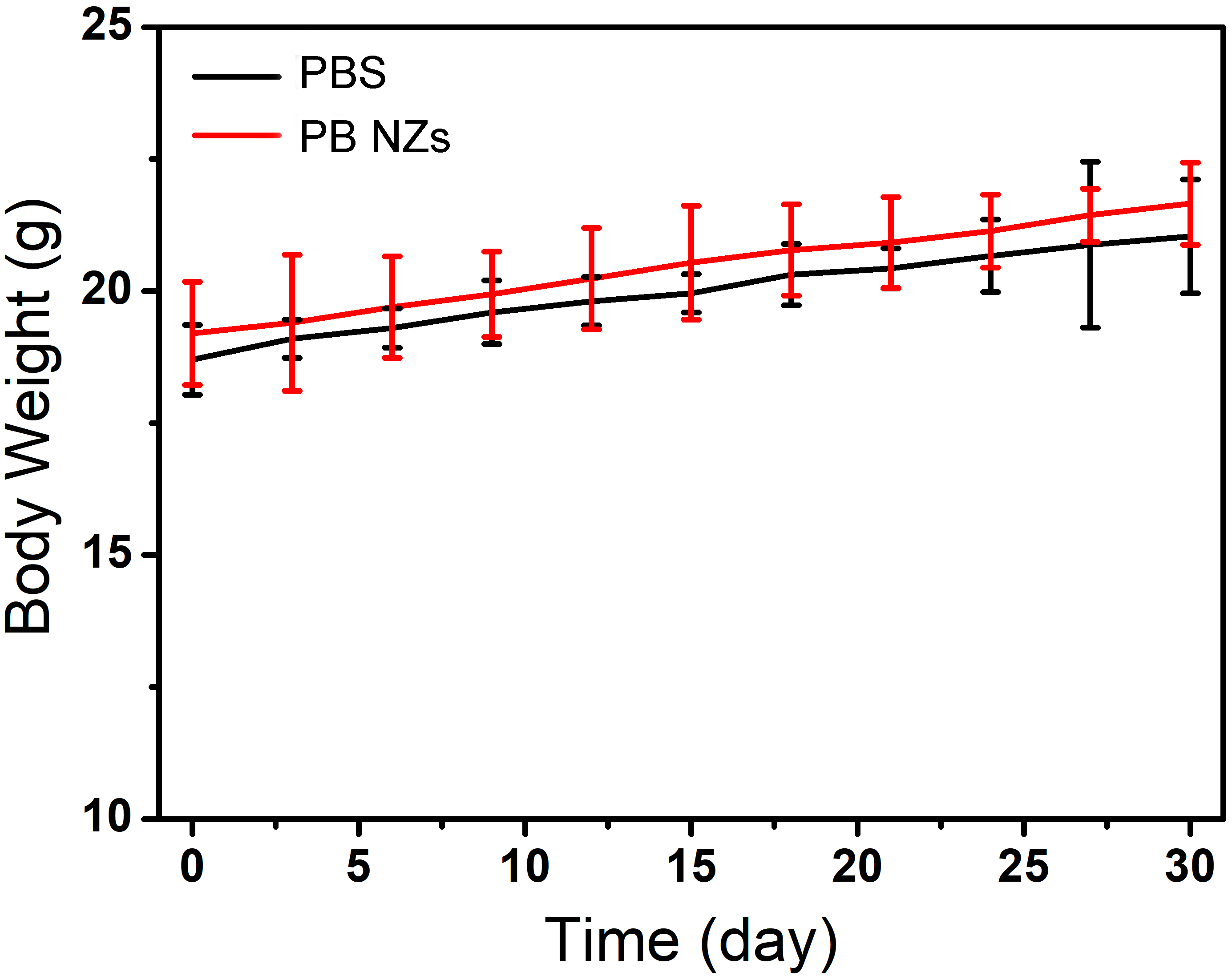


Figure S25. Body weight changes of mice with different treatments.

Table S1. The *in vivo* distribution of other reported nanomaterials and PB NZs.

|  | Cu_5.4_O USNPs^1^ | MMP^2^ | CeO_2_^3^ | Ir NPs-PVP^4^ | Pt NPs-PVP^5^ | **PB NZs** |
| --- | --- | --- | --- | --- | --- | --- |
| ID/g | 4.4 % | 11.3±2.3% | 7.8±3.4% | 18.7±4.6% | 13.5±2.9% | **6.3±1.6%** |

References

1. Liu T, Xiao B, Xiang F, Tan J, Chen Z, Zhang X, et al. Ultrasmall copper-based nanoparticles for reactive oxygen species scavenging and alleviation of inflammation related diseases. Nat Commun. 2020; 11: 2788.
2. Sun T, Jiang D, Rosenkrans ZT, Ehlerding EB, Ni D, Qi C, et al. A melanin based natural antioxidant defense nanosystem for theranostic application in acute kidney injury. Adv Funct Mater. 2019; 29: 1904833.
3. Zhang D-Y, Liu H, Li C, Younis MR, Lei S, Yang C, , et al. Ceria nanozymes with preferential renal uptake for acute kidney injury alleviation. ACS Appl Mater Interfaces. 2020; 12: 56830-8.
4. Zhang D-Y, Younis MR, Liu H, Lei S, Wan Y, Qu J, et al. Multi-enzyme mimetic ultrasmall iridium nanozymes as reactive oxygen/nitrogen species scavengers for acute kidney injury management. Biomaterials. 2021; 271:120706.
5. Zhang D-Y, Liu H, Younis MR, Lei S, Yang C, Lin J, et al. Ultrasmall platinum nanozymes as broad-spectrum antioxidants for theranostic application in acute kidney injury. Chem Eng J. 2021; 409: 127371.
